# Context-Dependent Dual Role of SKI8 Homologs in mRNA Synthesis and Turnover

**DOI:** 10.1371/journal.pgen.1002652

**Published:** 2012-04-12

**Authors:** Eavan Dorcey, Antia Rodriguez-Villalon, Paula Salinas, Luca Santuari, Sylvain Pradervand, Keith Harshman, Christian S. Hardtke

**Affiliations:** 1 Department of Plant Molecular Biology, University of Lausanne, Lausanne, Switzerland; 2 Lausanne Genomic Technologies Facility, Center for Integrative Genomics, University of Lausanne, Lausanne, Switzerland; Peking University, China

## Abstract

Eukaryotic mRNA transcription and turnover is controlled by an enzymatic machinery that includes RNA polymerase II and the 3′ to 5′ exosome. The activity of these protein complexes is modulated by additional factors, such as the nuclear RNA polymerase II-associated factor 1 (Paf1c) and the cytoplasmic Superkiller (SKI) complex, respectively. Their components are conserved across uni- as well as multi-cellular organisms, including yeast, Arabidopsis, and humans. Among them, SKI8 displays multiple facets on top of its cytoplasmic role in the SKI complex. For instance, nuclear yeast ScSKI8 has an additional function in meiotic recombination, whereas nuclear human hSKI8 (unlike ScSKI8) associates with Paf1c. The Arabidopsis SKI8 homolog VERNALIZATION INDEPENDENT 3 (VIP3) has been found in Paf1c as well; however, whether it also has a role in the SKI complex remains obscure so far. We found that transgenic VIP3-GFP, which complements a novel *vip3* mutant allele, localizes to both nucleus and cytoplasm. Consistently, biochemical analyses suggest that VIP3–GFP associates with the SKI complex. A role of VIP3 in the turnover of nuclear encoded mRNAs is supported by random-primed RNA sequencing of wild-type and *vip3* seedlings, which indicates mRNA stabilization in *vip3*. Another SKI subunit homolog mutant, *ski2*, displays a dwarf phenotype similar to *vip3*. However, unlike *vip3*, it displays neither early flowering nor flower development phenotypes, suggesting that the latter reflect VIP3's role in Paf1c. Surprisingly then, transgenic ScSKI8 rescued all aspects of the *vip3* phenotype, suggesting that the dual role of SKI8 depends on species-specific cellular context.

## Author Summary

The production and turnover of messenger RNAs (mRNAs) are conserved processes in eukaryotes, from single-cell organisms to plants and mammals. To some degree, this is also true for modulators of these processes, such as the Paf1 and SKI complexes. One particular protein, SKI8, has been described to have a role in the SKI complex, which influences mRNA stability, both in yeast and in mammals. Moreover, in yeast SKI8 has an additional role in meiotic recombination, whereas in humans it influences mRNA production through association with the Paf1 complex. This functional divergence is commonly thought to arise from differences in protein sequence between the yeast and mammalian SKI8 homologs. Here we show that the conserved SKI8 homolog of the model plant Arabidopsis acts in the SKI complex as well as the Paf1 complex, similar to human. However, using an Arabidopsis *ski8* mutant as a tool, we show that yeast SKI8 can fulfill all roles of Arabidopsis SKI8 if introduced into Arabidopsis cells. Thus, it appears that the functional divergence of SKI8 homologs might *a priori* be related to species-specific cellular context rather than divergence in protein sequence.

## Introduction

Production and turnover of eukaryotic mRNAs are highly conserved processes, which are mainly driven by RNA polymerase II (RNAPolII) and the 3′ to 5′ exosome (exosome), respectively [1], [2]. Regulation of transcription initiation by RNAPolII through promoter sequence-specific transcription factors is a major topic in developmental biology, since it is considered the prime mechanism for differential, cell and organ type-specific gene expression [3]. However, generic accessory factors, which are typically heteromultimeric protein complexes, exist as well. Compared to the RNAPolII machinery, they are less conserved but have been found in all uni- and multicellular eukaryotes investigated so far. In line with their lower conservation, these factors are generally not essential. However, loss of function mutations in their subunits typically result in pleiotropic phenotypes with varying degrees of severity. An example is the Mediator complex, which typically comprises more than 15 subunits and interacts with the C-terminal domain of the largest RNAPolII subunit [4], [5]. In yeast (*S. cerevisiae*), Mediator is associated with constitutively transcribed genes [6] and yeast Mediator mutants are typically viable but display impaired growth [4]. In multicellular organisms, the composition of Mediator is even more complex and individual subunit loss of function can lead to rather specific phenotypes. For instance, in the model plant Arabidopsis (*A. thaliana*), in which several additional Mediator subunits have been identified [7], respective mutants display such diverse phenotypes as increased cell proliferation, shifts in embryonic patterning or early flowering [7], [8], [9].

Screens for early flowering mutants also identified Arabidopsis subunit homologs of another conserved multimeric regulator of transcription, the RNAPolII-associated factor 1 complex (Paf1c) [10], [11], [12]. In yeast, Paf1c consists of five subunits [13], whose Arabidopsis homologs are *VERNALIZATION INDEPENDENCE* (*VIP*) *4*, *VIP5*, *EARLY FLOWERING* (*ELF*) *7*, *VIP6/ELF8* and *PLANT HOMOLOGOUS TO PARAFIBROMIN* (*PHP*) [10], [14], [15], [16]. Among the respective loss of function mutants, *php* mutants only flower early, whereas *vip4*, *vip5*, *elf7* and *vip6/elf8* mutants all display additional pleiotropic growth defects and aberrant flower development (e.g., variable floral organ number). The early flowering phenotype of *vip/elf* mutants has been linked to down-regulation of the central flowering time regulator, *FLOWERING LOCUS C* (*FLC*), via an epigenetic mechanism, consistent with a role of Paf1c in chromatin modification through changing histone methylation patterns [10], [11], [14]. The latter could also explain the phenotypes in flower development, which can be altered by mutation in epigenetic regulators [17].

Another mutant with dwarf, early flowering and aberrant flower development phenotypes is *vip3*. *VIP3* encodes a WD40 repeat protein, which is the putative Arabidopsis homolog of the yeast Superkiller (Ski) 8 gene [12]. SKI8 is part of the cytosolic SKI complex, which is thought to positively regulate exosome activity [1], [18], [19]. The SKI complex consists of a SKI8 dimer and the SKI2 RNA helicase, which are connected by their mutual interaction with the scaffold protein SKI3 [20]. Interestingly, human hSki8 as well as VIP3 also associate with Paf1c [11], [21], which is not the case for yeast ScSki8 [21], [22]. Rather, ScSki8 has a SKI complex-independent nuclear function in meiotic recombination [23]. This feature is not conserved in VIP3 [24], suggesting that Ski8 activity in plants might be functionally closer to mammals than unicellular eukaryotes.

Compared to its well documented role in Paf1c, the potential role of VIP3 in the SKI complex has not been characterized. Notably, although VIP3 is the top hit in a homology search of the Arabidopsis proteome using ScSki8 as a query, the next best hits are nearly equally significant with better overall coverage and represent structurally similar WD40 repeat proteins. Conversely, if the yeast proteome is queried with VIP3, more than two dozen hits score markedly better than ScSki8. Interestingly, the top hits, like PRP4 or TUP1, have been described as modifiers of pre-mRNA processing or chromatin modifications, respectively [25], [26], which would also be consistent with existing experimental data on VIP3 activity. However, reciprocal BLAST searches with higher eukaryotes clearly identify the respective SKI8 homologs as best hits. Still, experimental evidence for VIP3 involvement in the Arabidopsis SKI complex and the facets of the *vip3* phenotype that could be attributed to this role is missing. In this study, we investigate this question by a combination of biochemical, genetic and high throughput techniques.

## Results

### The Arabidopsis *zwg* mutant displays pleiotropic growth defects

**Figure 1:**
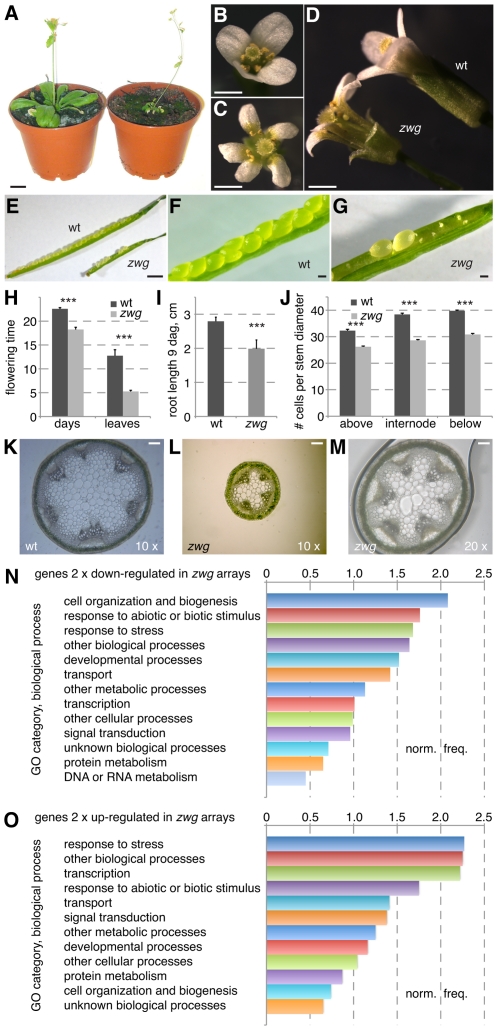
Phenotypic analysis of the *zwg* mutant. (A) Comparison of adult wild type Sav-0 plant (left) and *zwg* mutant (right). (B–D) Comparison of wild type (B) and *zwg* (C) flowers from different perspectives, note increased organ number and shorter organs in *zwg*. (E–G) Comparison of wild type and *zwg* siliques. (H) Comparison of flowering time measured as age or rosette leaf number. (I) Comparison of primary root length in tissue culture at 9 days after germination (dag). (J) Comparison of cell number per cell diameter measured at, above or below the 1^st^ internode. (K–M) Comparison of transverse stem sections taken below the first internode. (N–O) Gene ontology (GO) classification of genes down-regulated (N) or up-regulated (O) in *zwg* as compared to wild type, expressed as normalized frequency. Size bars are 1 cm (A), 1 mm (B–G) and 100 µm (K–M).

Analysis of natural genetic variation has become a common tool for isolation of allelic variants in Arabidopsis, facilitated by availability of collections of wild strains, so-called accessions. In the Slavice-0 (Sav-0) accession, we found largely infertile dwarf plants segregating at low frequency when grown in permanent light conditions and low humidity (∼40%) (Figure 1A). Moreover, careful investigation of the segregating population revealed a substantial fraction of non-viable, seedling lethal individuals. At higher humidity (∼60%) and long day conditions, the fraction of seedling lethals decreased considerably, whereas the ratio of dwarfs increased to near Mendelian (typically>20%) proportion, suggesting that the two classes represent the phenotypic spectrum of the same underlying genetic cause. The infertility of the dwarf plants, which rarely produced seeds and if so, very little (Figure 1E–1G), could be overcome to some degree by out-crossing with pollen from wild type looking plants. This allowed us to generate a segregating F2 population derived from a cross to the standard lab accession, Columbia-0 (Col-0). Genetic mapping revealed that the dwarf and infertility phenotypes segregated as a recessive single Mendelian locus on the lower arm of chromosome 4, which we named *ZWERGERL* (*ZWG*, Bavarian for “dwarf”).

A detailed analysis of the *zwg* phenotype revealed various floral defects. These included a low penetrance aberrant floral organ number phenotype (Figure 1B–1C) and shorter sepals (Figure 1D). Shorter anthers with few viable pollen accounted for the decreased fertility. The floral phenotypes were accompanied by early flowering, which was evident both in terms of age and rosette leaf number (Figure 1H). By following the development of individual seedlings from germination in tissue culture onwards, we could also detect reduced root elongation in *zwg* plants (Figure 1I). Radial growth of all organs was affected as well, as exemplified by the dramatically reduced diameter of the main inflorescence stem (Figure 1J–1M). Since the relative decrease in cell number (∼75% of wild type) (Figure 1J) was not as strong as the overall decrease in diameter (∼50% of wild type) (Figure 1K–1M), the reduced organ size in *zwg* mutants likely represents a combination of impaired cell proliferation and expansion.

Complementing the morphological characterization, we also analyzed the *zwg* transcriptome by hybridization of CATMA microarrays [27] with cDNA prepared from aerial tissues. Based on four replicate hybridizations, statistically solid expression changes (≥2-fold; p≤0.05) were found for 173 genes that were up-regulated and 425 genes that were down-regulated in *zwg* as compared to wild type (Table S1). These gene lists did not point to any specific defect in *zwg* mutants, such as mis-regulation of a particular hormone pathway. Rather, the genes represented an overall balanced sample across functional categories as illustrated by their gene ontology analysis (Figure 1N–1O). The only consistently over-represented category in both the up- and down-regulated sets was response to stress. In summary, our morphological as well as molecular characterization suggests that a general growth defect is responsible for the panoply of *zwg* mutant phenotypes.

### The *zwg* mutation is a novel null allele of *VIP3*

**Figure 2:**
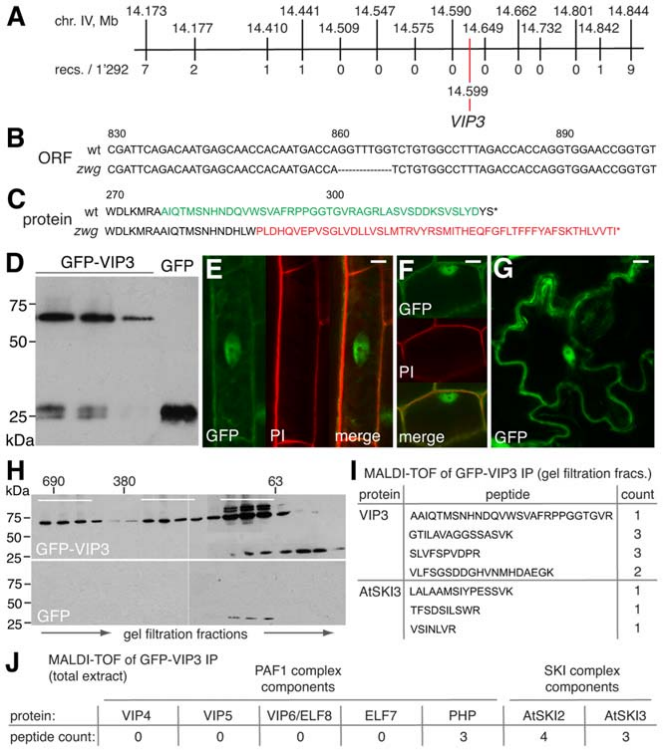
Isolation and characterization of the *zwg* locus. (A) Representation of the final mapping interval for *zwg*, including position of markers and the *VIP3* polymorphism. (B) Illustration of the 7 bp deletion in the *vip3^zwg^* coding sequence. (C) Conceptual translation of the *VIP3* wild type and *zwg* C-terminus. (D) Immunoblot analysis of transgenic GFP-VIP3 or GFP control lines, probed with anti-GFP antibody. Different independent lines are shown, GFP-VIP3 migrates below 75 kDa marker as expected. (E–G) Subcellular localization of GFP-VIP3 fusion protein in nucleus and cytoplasm of differentiated (E) or meristematic (F) root cells, and epidermal leaf cells (G). (H) Gel filtration analysis of GFP-VIP3, fractions pooled for subsequent co-immunoprecipitation of GFP-VIP3 are indicated by bars. (I) VIP3 and AtSKI3 (At1g76630) peptides identified in MALDI-TOF of co-immunoprecipitates obtained from the smallest set of fractions (H). (J) Number of peptides from Paf1c and SKI complex components identified in MALDI-TOF of co-immunoprecipitates obtained from total protein extract of GFP-VIP3 plants. Size bars are 10 µm (E–G).

To identify the molecular cause of the *zwg* mutation, we sequenced the genomes of Sav-0 wild type and *zwg* individuals with short reads [28]. Mapping of the reads onto the Col-0 reference genome revealed an extended region of heterozygosity on the lower arm of chromosome 4 in Sav-0 that encompassed the *ZWG* locus. The sequence information was exploited to generate polymorphic molecular markers that allowed mapping of the *zwg* mutation in the *zwg* x Col-0 population (Figure 2A). Within the zero recombination mapping interval, the sequence reads indicated the presence of a homozygous 7 bp deletion in the coding sequence of At4g29830, previously described as *VIP3*, in *zwg* but not in wild type (Figure 2B), which was confirmed by Sanger sequencing of respective PCR fragments. Analysis of a cross between *zwg* and a *vip3* null mutant (SALK_083364) [29] indicated non-complementation, confirming that *zwg* is indeed a new *vip3* allele, which we thus named *vip3^zwg^*. The deletion in *vip3^zwg^* encompasses nucleotides 861–867 of the open reading frame of the mRNA, which is expressed at similar levels in *vip3^zwg^* and wild type. Conceptual translation predicts that the deletion causes a frameshift to produce a 36 kDa instead of a 32 kDa protein with a modified and extended C-terminus, thereby disrupting the last of the five WD40 repeats of VIP3 (Figure 2C). Because of its phenotypic resemblance with the knock out allele, including the down-regulation of *FLC* expression (Table S1), and its recessive behavior, *vip3^zwg^* can be considered a null allele.

### VIP3 localizes to both nucleus and cytoplasm and is present in more than one protein complex

To clarify whether *VIP3* is indeed the functional Arabidopsis SKI8 homolog, we sought to determine its subcellular localization. To this end, we created a binary construct for expression of a *GFP-VIP3* fusion under control of the constitutive *35S* promoter. This transgene was introduced into Sav-0 wild type-looking plants that were heterozygous for the *zwg* mutation as determined by genotyping of the 7 bp deletion on high resolution agarose gels. Western analysis revealed variable expression of GFP-VIP3 fusion protein of the expected size in several independent lines (Figure 2D), within one order of magnitude of the level of endogenous *VIP3* as judged from qPCR. In the progeny of these plants, the *zwg* phenotype segregated in a proportion close to 1/16^th^ rather than 1/4^th^ and was significantly different from the segregation in the parallel grown non-transgenic mother line (Chi-square = 11.54 for df = 1, significant at p<0.001). None of the plants with a *zwg* phenotype carried the transgene as determined by genotyping. All other plants appeared wild type, suggesting that the fusion protein is functional and rescues all *zwg* phenotypes. Confocal microscopy showed both cytoplasmic and nuclear (but not nucleolar) localization of GFP-VIP3, in differentiated as well as proliferating cells, in both root and shoot tissues (Figure 2E–2G). Matching the dual subcellular localization, analysis of protein extracts by gel filtration detected the presence of GFP-VIP3 in at least two peaks, one in the ∼690 kDa and another in the 300 kDa range (Figure 2H). Moreover, substantial amounts were observed in smaller (100–200 kDa) fractions. To determine whether any of these fractions could represent the SKI complex or its sub-components, we collected three distinct sets of fractions after gel filtration and performed immunoprecipitations with anti-GFP antibody. Subsequent MALDI-TOF identified peptides of the Arabidopsis SKI3 homolog (At1g76630) in the pool of the smaller fractions (Figure 2I). Notably, protein homology searches unequivocally identify At1g76630 and ScSki3 as unique reciprocal and highly significant hits, suggesting that At1g76630 represents indeed the Arabidopsis SKI3 homolog. No other SKI complex or Paf1c components were identified, which might have resulted from our stringent conditions combined with previous gel filtration. Direct immunoprecipitation from total protein extract using the same conditions indeed not only identified AtSKI3, but also AtSKI2 (At3g46960, see below) and the Paf1c component PHP (Figure 2J). Thus, our analyses suggest that VIP3 is not only part of Paf1c in the nucleus, but also of the cytoplasmic SKI complex and likely represents the true SKI8 homolog.

### Random-primed high-throughput RNA sequencing suggests a role of VIP3 in mRNA turnover

To corroborate the consequent notion that *VIP3* should have a role in mRNA turnover, we applied a high throughput sequencing strategy to RNA samples isolated from *vip3^zwg^* and wild type. Because we aimed to sequence both full length mRNAs and mRNAs undergoing (3′ to 5′) degradation, cDNA from these samples was produced by random-primed rather than poly-T-primed synthesis. Prior to this, mRNA was enriched by removing the bulk of ribosomal RNA with the help of capture columns. The cDNA was then size-fractionated and the 200 bp fraction was used for preparation of the library, which was sequenced to produce single reads of 75 bp (21.3 mio. for wild type; 25.2 mio. for *vip3^zwg^*). The reads were mapped onto the Col-0 reference transcriptome, including the 5′ and 3′ UTRs, with relaxed stringency to accommodate nucleotide polymorphisms between Sav-0/*vip3^zwg^* and Col-0 [28]. Parallel mapping onto the Col-0 reference genome placed the large majority of reads in exons (80.2% in wild type; 84.5% in *vip3^zwg^*), confirming that our sequence data represent RNA molecules and that genomic contamination, if any, is negligible (Table S2). For the follow up analyses, we concentrated on the reads that mapped onto mRNA (27.0% in wild type; 13.8% in *vip3^zwg^*), and in particular on the nuclear encoded genes (16.7% of reads in wild type; 6.6% in *vip3^zwg^*).

**Figure 3:**
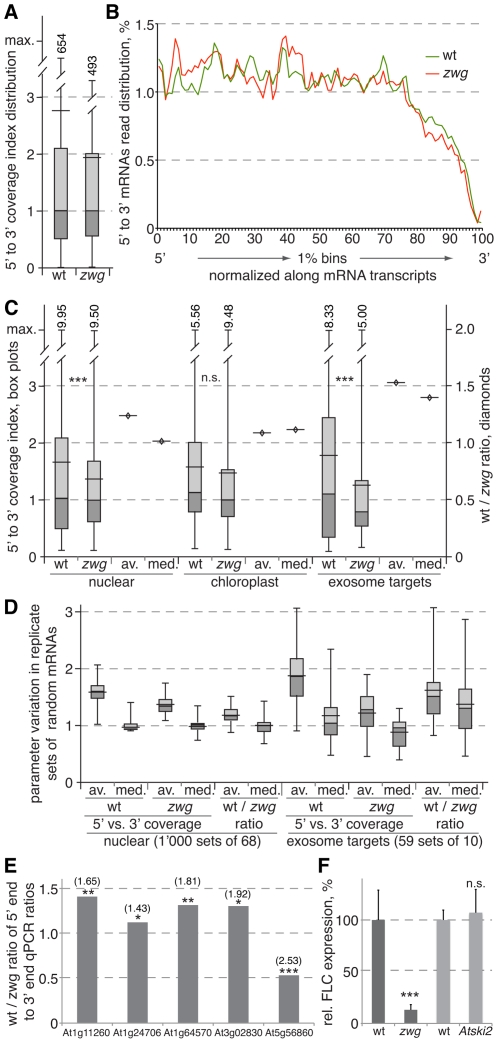
Analysis of random-primed RNA sequencing of wild-type and *vip3^zwg^* mRNAs. (A) Distribution of the 5′ to 3′ coverage index (i.e. number of reads mapping into the 20% 5′-most region divided by number of reads mapping into the 20% 3′-most region of a transcript) in wild type and *vip3^zwg^*. The box plots indicate maximum, minimum, median and quartile values, as well as the average (wider horizontal bar). Out of range maximum values were added numerically. (B) Relative read abundance in 1% bins cumulated for the 10% of most highly expressed transcripts (n = 2’437). (C) Distribution of the 5′ to 3′ coverage index (box plots, left y axis) and the ratios of median and average between wild type and *vip3^zwg^* (diamonds, right y axis) in nuclear (n = 5’617), chloroplast (n = 34) and prime exosome target (n = 68) genes. Maximum values were added numerically. (D) Distribution of the averages and medians of the 5′ to 3′ coverage index and their wild type to *vip3^zwg^* ratio in random sets drawn from the nuclear or prime exosome target sets. (E) Wild type to *vip3^zwg^* ratio of the 5′ to 3′ end ratios for selected genes as determined by qPCR analyses of three independent RNA samples for each genotype. A value >1 indicates stabilized transcript in *vip3^zwg^*. The respective 5′ to 3′ coverage ratios from the RNA sequencing are given in brackets above. (F) Relative expression levels of *FLC* in wild type, *vip3^zwg^* and *Atski2*. n.s.: not significant; *: p<0.05; **: p<0.01; ***: p<0.001.

In total, of 26’598 transcripts interrogated, 14’228 were covered by at least one read in both the wild type and *vip3^zwg^* sample. In order to obtain a parameter that would allow us to estimate the steady state abundance of full length versus degrading mRNAs, we calculated the ratio between the number of reads mapping onto the 5′-most 20% of a transcript versus those mapping onto the 3′-most 20%. After removal of nonsense values (i.e. 0 or ∞ because of absent coverage of one end) and outliers with extreme values (resulting from excess read abundance combined with obvious mis-mapping, e.g. reads covering the flanking region of a gypsy-like retrotransposon [At4g06477]), the distribution of this 5′ to 3′ coverage index was skewed towards values >1. To some degree this likely represents a technical bias [30], but could also reflect a dominant role of 3′ to 5′ degradation in mRNA turnover. Interestingly, the 5′ to 3′ coverage index was generally higher in the wild type than in the *vip3^zwg^* sample (Figure 3A). To verify that this was not a technical artifact, we compared the relative proportion of the accumulated reads in 1% bins along the 10% most highly expressed nuclear encoded transcripts of wild type. The respective profiles for the wild type and *vip3^zwg^* sample were similar (Figure 3B), suggesting that the RNA sequencing data from the two samples are comparable.

### Prime exosome target transcripts are significantly stabilized in *vip3^zwg^*

In order to remove statistically doubtful 5′ to 3′ coverage index values that were due to low transcript abundance, we only considered the 6’500 transcripts for which at least 50% of sequence was covered in both the wild type and *vip3^zwg^* samples in follow up analyses. Remaining outliers with index values ≥10 or ≤0.1 were removed as well. From this set, we extracted the group of 5’617 nuclear encoded transcripts that were not strongly affected by depletion of exosome activity [31], as well as 34 chloroplast-encoded transcripts and 68 nuclear-encoded transcripts that were significantly stabilized upon exosome depletion and can be considered prime exosome targets [“the hidden transcriptome”; 31] (Table S3). The 5′ to 3′ coverage index value distribution in the nuclear control group confirmed the earlier picture of higher overall values in wild type as compared to *vip3^zwg^*, which is for instance also evident in the ratios between the respective averages or medians (Figure 3C). While no significant difference was found in the chloroplast transcripts, this trend was amplified in the prime exosome targets, which displayed higher average and median index values in wild type and lower ones in *vip3^zwg^* as compared to the nuclear control group (Figure 3C).

To evaluate the robustness of the difference between the nuclear control group and the exosome targets, we determined the index value distribution for 1’000 random sets of 68 genes extracted from the nuclear control group. These analyses confirmed the trend towards higher values in wild type, underlined by the finding that the wild type to *vip3^zwg^* ratio of averages and medians was nearly always >1 (Figure 3D). Notably, even the maximum ratios observed within the 1’000 sets did not or barely reach the values observed in the exosome target set (1.50 versus 1.53 for the average, 1.40 versus 1.39 for the median). Conversely, within 59 random sets of 10 transcripts extracted from the exosome targets, the trend towards higher values in wild type and lower ones in *vip3^zwg^* including the ratios was always evident (Figure 3D). We confirmed this finding by an independent method with independent, triplicate RNA preparations for a set of five randomly chosen genes. For each gene, oligonucleotide pairs for qPCR detection of the respective 5′ and 3′ mRNA ends were designed. The reverse primers for each fragment were used to prime separate cDNA synthesis reactions. Subsequent qPCR allowed quantification of the 5′ and 3′ end abundance for each gene in the replicate samples of the two genotypes. With one exception, the ratio between the 5′ and 3′ end abundance was always higher in wild type than in *vip3^zwg^* mutants, as expressed by the ratio between those ratios being greater than 1 (Figure 3E). In summary, these analyses suggest that nuclear encoded mRNAs in general and prime exosome targets in particular are stabilized in *vip3^zwg^* mutants.

### *Atski2* mutants display a dwarf, but no flowering or flower development phenotype

**Figure 4:**
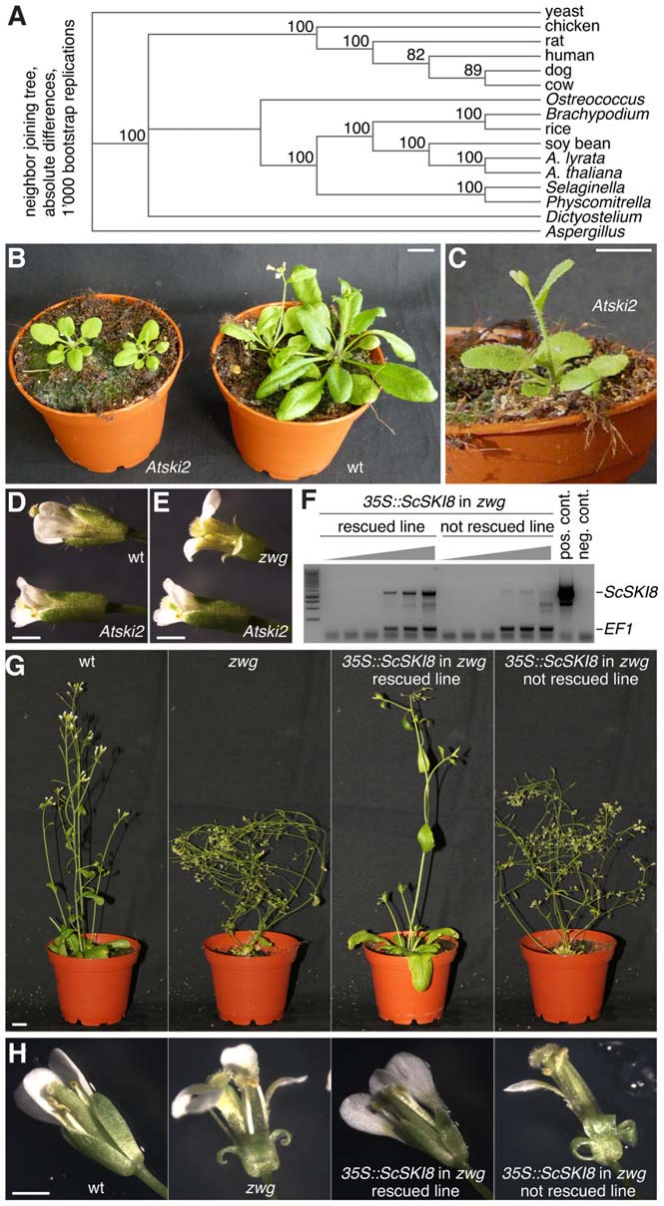
Phenotypes of the *Atski2* mutant and *vip3^zwg^* rescued by transgenic ScSki8 expression. (A) A phylogenetic tree of SKI2 homologs from various eukaryotic species including AtSKI2. (B) Comparison of adult wild type Col-0 plants (right) and *Atski2* mutants (left). (C) Close-up of an *Atski2* mutant. (D–E) Comparison between wild type, *Atski2* and *vip3^zwg^* mutant flowers at anthesis. (F) Semi-quantitative RT-PCR (increasing number of amplification cycles) of transgenic ScSki8 expression in *vip3^zwg^* plants as compared to the elongation factor 1 gene (*EF1*). (G–H) Illustration of the rescue of dwarf (G) and flower development (H) phenotypes by transgenic expression of ScSki8 in *vip3^zwg^* mutants. Size bars are 1 cm (B, C, G) and 1 mm (D, E, H).

To determine which aspects of the phenotype spectrum of *vip3* mutants are due to its involvement in Paf1c or the SKI complex, respectively, we sought to characterize mutants in other SKI subunit homologs of Arabidopsis. Whereas knock out mutants in the SKI3 homolog were not available in reverse genetic collections [29], a line segregating a T-DNA insertion in exon 9 out of 23 of At3g46960 (SALK_118579) was available. Similar to At1g76630 and ScSki3, reciprocal homology searches between Arabidopsis and yeast using ScSki2 or At3g46960 as a query identified each other as the uncontested top hits, suggesting that At3g46960 represents the unique ScSki2 homolog in Arabidopsis (*AtSKI2*). This notion is also supported by a phylogenetic analysis (Figure 4A; Text S1). Analysis of the SALK_118579 line revealed that it segregates up to ∼25% of dwarf plants (Figure 4B–4C). This phenotype co-segregated perfectly with homozygosity of the T-DNA insert and absence of full length *AtSKI2* mRNA. With the caveat that residual RNA production 3′ from the T-DNA insertion site has been reported previously [32], the SALK_118579 line therefore might represent the *Atski2* null mutant phenotype. Contrary to the *vip3* mutants however, the dwarf phenotype was neither accompanied by a flower development nor an early flowering phenotype (Figure 4D–4E). In line with the latter observation, *FLC* expression was strongly diminished in *vip3^zwg^*, but not in *Atski2* mutants (Figure 3F). In summary, these observations suggest that the flower development and early flowering phenotype of *vip3* mutants could reflect *VIP3's* role in Paf1c rather than the SKI complex.

### *ScSki8* rescues all aspects of the vip3^zwg^ phenotype

Considering that ScSki8 has not been found to associate with Paf1c, we sought to corroborate this notion by testing whether transgenic ScSki8 could rescue the dwarf phenotype of *vip3^zwg^* mutants. To this end, the *ScSki8* open reading frame was cloned into a binary construct for constitutive expression under control of the *35S* promoter. Again, the transgene was introduced into Sav-0 wild type plants that were heterozygous for the *vip3^zwg^* mutation. Genotyping of the 7 bp deletion and the transgene in the segregating progeny identified several homozygous *vip3^zwg^* mutants carrying the *35S::ScSki8* transgene. These plants developed either as dwarf or as wild type, and this was correlated with transgene expression (Figure 4F–4G). Thus, transgenic expression of ScSki8 could rescue the dwarf phenotype of *vip3^zwg^* mutants. Moreover, surprisingly both the early flowering and flower development phenotypes were also rescued (Figure 4H). Therefore, our data suggest that once introduced into Arabidopsis, ScSki8 can fulfill all functions of VIP3, including those not normally encountered in yeast itself.

## Discussion

In this study, we present experiments that lead to four main conclusions: First, we show that VIP3 is the *bona fide* SKI8 homolog of Arabidopsis; second, we demonstrate that next generation sequencing of random-primed RNA samples with short reads can be used to estimate the turnover of mRNA transcripts; third, we show that the phenotypic aspects of VIP3 function in Paf1c and the SKI complex can be separated; and fourth, we provide evidence that the dual role of SKI8 homologs in Paf1c and the SKI complex appears to depend on the species-specific cellular context.

Our interest in *VIP3* originates from the discovery of the *zwg* mutant that segregated in the Arabidopsis Sav-0 accession. It seems unlikely that the 7 bp deletion in *vip3^zwg^* represents indeed an allelic variant recovered from a natural environment because of its detrimental phenotypic consequences. A haplo-insufficient beneficial effect of *vip3^zwg^* could explain maintenance of the allele by balancing selection, however, we did not observe any obvious phenotypes in the heterozygous plants that would support this idea. Rather, it appears likely that *vip3^zwg^* is a spontaneous allele that has arisen during the propagation of the Sav-0 accession in stock centers starting in the 1960s over several decades [33], [34].

At the outset of our study, it was still unclear whether *VIP3* is indeed the Arabidopsis *SKI8* homolog. While its role in epigenetic regulation of *FLC* transcription through association with the Paf1c complex had been well documented [11], [14], its potential role in the SKI complex had not been characterized. Because of the evolutionary distance between higher plants, yeast and mammals this could not be considered a given, in particular as VIP3 and SKI8 fall into an abundant class of structurally similar WD40 repeat proteins. This was underlined by the finding that ScSki8 is by far not the closest VIP3 homolog in yeast. For instance, position-specific iterated BLAST identifies more than two dozen yeast proteins that are more homologous to VIP3 than ScSki8 (e.g., a 93.2 score, 69% coverage and 5×10^−24^ e-value for PRP4 as compared to 48.9 score, 40% coverage and 5×10^−8^ e-value for ScSki8). It is only our functional analyses that suggest that VIP3 is indeed the *bona fide* ScSki8 homolog. Consistent with a potential role in both Paf1c and the SKI complex, we found that VIP3 is present in both the nucleus and cytoplasm, and in at least two protein complexes of distinct size. The larger peak fractions around 690 kDA could represent Paf1c, whereas the peak around 300 kDa could represent the SKI complex [21]. A third peak around even smaller (100–200 kDa) size fractions could represent partial components of these complexes or VIP3 dimers, which might accumulate in excess as the *GFP-VIP3* transgenes were typically expressed at higher levels than endogenous *VIP3*. Interestingly, immunoprecipitation of GFP-VIP3 after gel filtration identified association with the Arabidopsis SKI3 homolog, but not the SKI2 homolog. This might mean that the SKI complex dissociates into sub-components during gel filtration and/or that SKI2 is lost during immunoprecipitation washes. Alternatively, it could reflect the fact that SKI8 interaction with SKI3 is direct, while interaction with SKI2 is indirect [20]. However, when directly immunoprecipitated from total protein extract, AtSKI3 as well as AtSKI2 was pulled down in our stringent conditions, underlining that VIP3 is indeed part of the SKI complex.

The notion that VIP3 is a functional subunit of the SKI complex is supported by our genome-wide analysis of mRNA stability in *vip3^zwg^* mutants. To estimate mRNA turnover was foremost a technical challenge, because it meant that standard cDNA synthesis using poly-T oligonucleotides directed against the 3′ poly-A tail of mRNAs could not be applied. This also abolished the inherent selection of the mRNA fraction for sequencing from the much larger amount of ribosomal or transfer RNAs. Instead, to also capture mRNAs undergoing 3′ to 5′ degradation, cDNA was synthesized with random-priming, and the mRNA fraction was enriched by removing ribosomal RNAs through capture columns. High throughput sequencing of the cDNA samples and subsequent read mapping onto the reference transcriptome revealed that our method efficiently enriched the mRNA fraction, which generally represents 1–2% in total RNA samples, about 5 to 10-fold. The relative read abundance along transcripts is to some degree determined by technical biases, such as the directionality of cDNA synthesis [30]. However, it should also reflect the steady state equilibrium between mRNA synthesis and breakdown considering that primers were not limiting in cDNA synthesis and that poly-A tails provide priming sites but are not included in the sequence analysis. Generally, the coverage profiles displayed a decrease from 5′ to 3′, suggesting that exosome-mediated 3′ to 5′ degradation is the main driver of mRNA breakdown [18], [35]. To quantify the stability of individual transcripts, we defined a 5′ to 3′ coverage index, which was generally >1, consistent with the overall profile. The comparison of the 5′-most 20% of a transcript versus its 3′-most 20% was designed to avoid skewed values in the case of poorly covered transcripts, and indeed comparatively few outliers were observed. In some cases, these reflected obvious mismappings because of repetitive or redundant sequences (e.g. retrotransposon borders), while in others mismapping might have occurred because of the relaxed stringency that was required to map mRNA sequences from a divergent accession onto the reference transcriptome [28]. Overall, the patterns as well as the quantitative difference between the wild type and *vip3^zwg^* samples were robust, even if more selective criteria were applied or if other indexes were considered, such as linear fitting of read coverage. Thus, the index values suggest that in the *vip3^zwg^* sample the relative abundance of intact 3′ ends as compared to 5′ ends is higher, pointing to a shifted steady state equilibrium between mRNA transcription and degradation. This finding is consistent with the generic role of the SKI complex in exosome activation [18] and was particularly evident in the group of the most prominent exosome targets, termed the “hidden transcriptome” [31]. In summary, our data support the idea that VIP3 is a SKI complex component that affects mRNA stability and that random-primed RNA-Seq is a valid approach to estimate mRNA turnover.

The implication of VIP3 in the SKI complex suggests that the *vip3* phenotype should reflect the combination of VIP3 function in both Paf1c and the SKI complex. The availability of a mutant in the *AtSKI2* gene, which can be unequivocally identified by homology searches, enabled us to disentangle the two activities. Interestingly, *Atski2* plants displayed dwarfism, but neither early flowering nor aberrant flower development. Thus, the latter aspects of the *vip3* phenotype should primarily result from impaired Paf1c function. It is noteworthy however that the *Atski2* dwarf phenotype is not as severe as in *vip3*, and that growth defects have also been observed in mutants of other Paf1c components. It thus appears likely that the SKI complex-related growth defects in *vip3* are aggravated by the additionally impaired Paf1c activity.

To clarify more directly which portions of the *vip3* phenotype are attributable to impaired Paf1c or SKI complex function, we sought to exploit the fact that ScSki8 does not associate with Paf1c in yeast [21], [22] and presumably also not in Arabidopsis. However, to our surprise ScSki8 was able to fully rescue all aspects of the *vip3* phenotype. Thus, it appears that in the cellular context of Arabidopsis, ScSKI8 can fulfill VIP3's role in Paf1c. This could mean that other factors determine whether SKI8 is recruited to Paf1c or not, and that in this sense Arabidopsis is closer to mammals than yeast. Indeed we also tried to complement *vip3^zwg^* by constitutive expression of the mouse SKI8 homolog, WDR61. However, for unknown reasons, we never managed to recover transgenic plants in repeated transformation attempts, which could mean that WDR61 expression is poisonous for Arabidopsis. Thus, while cellular context must play an important role, SKI8 function might to some degree also depend on inherent features. Future experiments to determine the interaction patterns of different SKI8 homologs and derivative point mutants of interest are a promising avenue to clarify this issue in detail.

## Materials and Methods

Molecular biology and genetics standard procedures, such as plasmid construction, genomic DNA isolation, qPCR, genotyping, or gene mapping were performed as described [36], [37].

### Plant materials, growth conditions, and phenotypic analyses

The Sav-0 accession used in this study has been described previously [28]. T-DNA insertion lines were obtained from the Nottingham Arabidopsis Stock Centre and the insertions in lines SALK_083364 [knock out of *VIP3* (At4g29830)] and SALK_118579 [knock out of *AtSKI2* (At3g46960)] were confirmed by PCR analysis. For propagation and analysis of lines, seeds were germinated on half-strength Murashige & Skoog media in tissue culture and transferred to soil at 10–12 days after germination. Plants were then grown in either permanent light and ∼40% humidity, or 16 hr light–8 hr dark cycles and ∼60% humidity at 22°C. The latter conditions were used for characterization of the phenotypes displayed in the figures. For determination of flowering time, seeds were germinated directly on soil and the number of days or of rosette leaves was scored on the first day when the inflorescence meristem became visible. For root growth measurements, seedlings grown vertically in tissue culture were scored at 9 days after germination using ImageJ software and then transferred onto soil to determine wild type or mutant phenotype in the adult shoot.

### Microscopy

For transverse sections, stem segments encompassing the first internode were cut with a razor blade and embedded in 6% agarose. From these samples, 85 µm sections were obtained using a Leica-VT 1000S vibratom and photographed using a Leica Diaplan 3 microscope. Subcellular localization of GPF-VIP3 was determined in shoots and roots of 6 day old seedling using a Zeiss LSM 510 confocal microscope. For the propidium iodide staining the roots were incubated for 2–5 min in a 50 µg/ml solution.

### Transgenic constructs

For expression of *VIP3* or ScSki8 under control of the *35S* promoter, the respective open reading frames amplified from cDNA samples were cloned into vectors pMDC43 or pMD32 [38], respectively. Construct integrity was verified by Sanger sequencing before transfer into Agrobacterium and transformation of Arabidopsis plants by the floral dip method. Constructs were introduced into Sav-0 wild type looking plants that were heterozygous for *vip3^zwg^* as determined by genotyping. For genotyping, PCR was performed on genomic DNA using oligonucleotides GAG CTG CGA TTC AGA CAA TGA G and GCC CGG ACA CCG GTT CCA C. The PCR products of 87 bp from the wild type or 80 bp from the *vip3^zwg^* allele were resolved on 4% agarose gels. Transformants were selected on hygromycin and homozygous *vip3^zwg^* plants among the transformants were selected by genotyping. Transgenic *GFP-VIP3* or ScSki8 expression levels were determined by quantitative real time or semi-quantitative RT-PCR, respectively, and normalized compared to the *EF1* gene as described [36].

### Microarray analyses

For microarray analysis, a mix of equivalent amounts of aerial tissues (rosette leaves, stems, cauline leaves, inflorescences) from 4 week old adult plants was collected and frozen in liquid nitrogen before total RNA was prepped using the QIAGEN RNeasy Plant Mini Kit. cDNA synthesis, labeling, hybridization onto CATMA microarrays [27] and data analysis was then performed as described previously [36].

### Random-primed high-throughput RNA sequencing

For RNA-Seq, rosette leaves were harvested from 25 day old phenotypically wild type or *vip3^zwg^* plants. Genomic DNA was isolated from one part of each sample to verify genotypes, whereas total RNA was prepped from the remaining tissue using a QIAGEN RNeasy Plant Mini Kit. Ribosomal RNA was subsequently largely removed by treating 10 µg of total RNA with Invitrogen RiboMinus Plant Kits following the manufacturer's instructions. The enriched mRNA samples were then subjected to random-primed cDNA synthesis, amplification, size selection and high throughput sequencing with 75 bp single reads on an Illumina instrument as described [28]. Read mapping onto the Col-0 reference genome or transcriptome (main gene models, TAIR 9.0 release) was performed using the BWA program [39] with a seed length of 50 bp and up to 5 mismatches or gaps allowed.

### Protein work

Total protein was extracted from *35S::GFP-VIP3* or *35S::GFP* transgenic plants as described before fractionation by gel filtration using an Amersham Superdex 200 10/300 GL FPLC column with a buffer flow rate of 0.5 ml/min [40]. Consecutive 0.5 ml fractions were collected, concentrated and subjected to 10% SDS–PAGE followed by protein immunoblot analysis. Fusion protein was detected using an anti-GFP antibody (dilution 1∶3000) (Living colors, Clontech). For co-immunoprecipitation of GFP-VIP3, three consecutive sets of gel filtration fractions were pooled and incubated for 90 min. at 4°C with 50 µl of μ-magnetic beads conjugated to anti-GFP antibody (μMACS anti-GFP MACS, Miltenyi Biotec). The slurry was passed through a magnetic column, washed 5 times with protein extraction buffer before elution of proteins with hot protein loading buffer. Samples were analyzed by immunoblot (anti-GFP) and silver staining prior to MALDI-TOF analysis. For MALDI-TOF, samples were migrated on a 12% mini polyacrylamide gel for about 2.0 cm, and rapidly stained with Coomassie blue. Entire gel lanes were excised into 5 equal regions from top to bottom and digested with trypsin (Promega) as described [41], [42]. Data-dependent LC-MS/MS analysis of extracted peptide mixtures after digestion with trypsin was carried out on a hybrid linear trap LTQ-Orbitrap XL mass spectrometer (Thermo Fisher Scientific) interfaced to a nanocapillary HPLC equipped with a C18 reversed-phase column (Agilent Technologies). Collections of tandem mass spectra for database searching were generated from raw data with Mascot Distiller 2.3.2, and searched using Mascot 2.3 (Matrix Science) against the 2011_03 release of the UNIPROT database (SWISSPROT+TrEMBL, www.uniprot.org), restricted to *Arabidopsis thaliana* taxonomy (50’756 sequences after taxonomy filter). Mascot was searched with a fragment ion mass tolerance of 0.50 Da and a parent ion tolerance of 10 ppm. The digestion enzyme trypsin was specified with one missed cleavage. Iodoacetamide derivative of cysteine was specified as a fixed modification. N-terminal acetylation of protein, deamidation of asparagine and glutamine, and oxidation of methionine were specified as variable modifications. The software Scaffold (version Scaffold_3.0.9, Proteome Software Inc.) was used to validate MS/MS based peptide (minimum 90% probability [43]] and protein [min 95% probability [44]) identifications, perform dataset alignment as well as parsimony analysis to discriminate homologous hits.

### qPCR analyses

To determine the abundance of 5′ and 3′ ends of selected mRNAs, total RNA was prepared from three independent wild type and *vip3^zwg^* replicate samples. Separate cDNA syntheses were performed for each individual gene fragment, followed by qPCRs that were performed as described [36] to detect the respective 5′ and 3′ ends of the transcripts. The following oligonucleotides were used: AT1G01010: GAC AGC TCA ACA CTT TTC CAC TTC and CTT TTA TCC TAA ACA AGA CCC GTA AAG (5′ end); GAA CGA AGC ATG TTT GAT TTA TCA TTG and TTG TTG GTG GTT CAT TGG AGT ACA (3′ end); AT1G24706: GTT CCT CTC CCT TTT CAT CTT ATC G and CAT CTT AAA CCC CTT TCG TGT GTA T (5′ end); GAT TTG CAG ATC CTT TGG TTT GTT C and GCT ATG AAT ATA TCT GAA GTC TGG CAA G (3′ end); AT1G64570: CCA TTT ATC GAT TCT TCA CAG ACA CG and GAT TTC ATG ACT CAA ATT AGG GTT CCA (5′ end); GAT GCT GAG GAT GAG TAA GTT CCT TC and GCT AGT AAT CTG CAT TCA AAC AGC ACT A (3′ end); AT3G02830: CAC TAC CTC TCA CCT CTC TGT TTA CAC and CCA TAG ACG TGA AGA GGA AGA ATG (5′ end); GAA GAA ACA AAG GAA GAA GAA GAA GAG and CCA TAG ACG TGA AGA GGA AGA ATG T (3′ end); AT5G56860: ATT GAT GAG ATA AAC AAA TGA AGA CAC AAA G and CCA TGT GTG TTT GGC TCG TGT C (5′ end); GTT GAT CAG ATC ATC ACA ATA TCC TCA TTA C and GCT ATT AAT TAT CAT ATT AAA CTC TCA CAC ACT CT (3′ end); For detection of *FLC* expression in relation to the *EF1* housekeeping gene, qPCR was performed as described [36] using the same oligonucleotide pairs.

## Supporting Information

Table S1 Up- and down-regulated genes in microarray experiments comparing *vip3^zwg^* mutants to their Sav-0 wild type background.
(XLS)

Table S2 Read number and coverage statistics for the RNA-seq experiments.
(XLS)

Table S3 RNA-seq read coverage of individual mRNAs and their deduced 5′ to 3′ coverage indices in *vip3^zwg^* mutants and the Sav-0 wild type background.
(XLS)

Text S1 Supplemental alignment. Alignment of the protein sequences of various SKI2 homologs from different species.
(PDF)
