# Supplementary material for: Context-Dependent Dual Role of SKI8 Homologs in mRNA Synthesis and Turnover
Source: PLoS Genet. 2012 Apr 12;8(4):e1002652. doi: 10.1371/journal.pgen.1002652 (PMC3325215; doi:10.1371/journal.pgen.1002652)
Supplement: Text S1 — Supplemental alignment. Alignment of the protein sequences of various SKI2 homologs from different species. (PDF) [file pgen.1002652.s004.pdf]

Formatted Alignments

```
                                                    10                  20                  30
Ostreococcus tauri            - - - - - - - - - - - - - - - M K R K A C E E V G I A S F H C E L P G T A
Canis lupus                   - - - - - - - M M D T E R L A L P P P D P L D L P L R P V E L G C T G R W
Saccharmyces cerevisiae       - - - - - - - - - - - - - - M S E G F S S S S I Q E L Y Q S L K E I T N N
Aspergillus nidulans          - - - - - - - - - - - - - - M A E T V A S I L V D L N L K T E N L S G S A
Bos taurus                    - - - - - - - M M E T E R L V L P P P D P L D L P L R A V E L G C T G R W
Homo sapiens                  - - - - - - - M M E T E R L V L P P P D P L D L P L R A V E L G C T G H W
Pan troglodytes               - - - - - - - - - - - - - - - - - - - - - - - - - - - - - - - - - - - - -
Dictyostelium discoideum      M T T T V Q D I Q D F L R E L E G L E K E S T G E L A A S T P S H L V Y N
Rattus norvegicus             - - - - - - - M M E T E R L V L P P P D P L N L P L R A L E V G C T G R W
Selaginella moellendorfii     - - - - - - - - - - - - - - M A P P K L G G E L P F K I G V A G Y S G E L
Physcomitrella patens         - - - - - - - - - - - - - - M A P - R L G G D V P F K L S L A G Y G G N L
Brachypodium distachyon       - - - - - - - - - - - - - - - - - - - - - - - - - - - - - - - - - - - - -
Oryza sativa                  - - - - - - - - - - - - - - - - - - - - - - - - - - - - - - - - - - - - -
Glycine max                   - - - - - - - - - - - - - - M D P I Q A A N E L A F R V G F S G H S G H L
Arabidopsis lyrata            - - - - - - - - - - - - - - M N R V Q A G N E L G F R V G F S G H G G H L
Arabidopsis thaliana          - - - - - - - - - - - - - - M N K V E A G N E L G F R V G F S G H G G H L
                              M T T T V Q D M M E T E R L M   P           . L     R .       G     G
```

```
                                                    70                  80                  90
Ostreococcus tauri            A N - - - - - - - - - - - - - - - V H G - - I L V Q L P L P K G V N E E R V
Canis lupus                   H G L - - - - - - - P P - C A P D L Q Q E A E Q L F L S S P A W L P L H G
Saccharmyces cerevisiae       D E - - - - - - - - P K - - - - H A N D I I K D R F L R P S N A L P W S L
Aspergillus nidulans          R Q R - - - - - - - P R Q S A S D L K D E L E K E F L T P S P R F S P E W
Bos taurus                    H G L - - - - - - - P P - C A P D L Q Q E A E R L F L S S P A W L P L H G
Homo sapiens                  H G L - - - - - - - P P - C A P D L Q Q E A E Q L F L S S P A W L P L H G
Pan troglodytes               - - - - - - - - - - - - - - - - - - - - - - - - - - - - - - - - - - - - -
Dictyostelium discoideum      D N N D F S L F N F P I S S N V T I P K Q N N Q Q D I K K E L S Q Q Y I N
Rattus norvegicus             H G L - - - - - - - P P - C A P D L C Q E A E Q L F L S S P A W L P L H G
Selaginella moellendorfii     P V Q - - - - - - - P P Q T S A G I L A E I E E K Y L V P R L D R E I D D
Physcomitrella patens         P P F - - - - - - - P P E T R E S A L R K I E E Q Y L T P C L D E A A S D
Brachypodium distachyon       - - - - - - - - - - - - - - - - - - - - - - - - - - - - - - - - - - - - -
Oryza sativa                  - - - - - - - - - - - - - - - - - - - - - - - - - - - - - - - - - - - - -
Glycine max                   P A F - - - - - - - P S E T P E S I K K Y I E E T Y L Q P R L D P D D F S
Arabidopsis lyrata            P A F - - - - - - - A K E T K E S I K K H I E E K Y L L P R L E P D Q F S
Arabidopsis thaliana          P A F - - - - - - - A K E T K E S I K K H I E E K Y L I P R L E P D Q F S
                                    D F S L F N F P   E         .       I E     . L   P   .
```

```
  40                  50                  60
T E E E I L K T V R - - - - - - - - R F N A D
E L L N V P G A - - - - - - - - - P E S T L P
A D V E L F E D R I T K - - - - - L D F E S T
F D A Q L A E E E K G S - - - - - Y R H R G P
E L L N V P G A - - - - - - - - - P E S T L P
E L L N L P G A - - - - - - - - - P E S S L P
- - - - - - - - - - - - - - - - - - - - - - -
N D K S L K T N I T A S G P N S I P D L R V F
E L L N V P G P - - - - - - - - - P E S T L P
Q V E I L P S I S R S N P L D D L P E L I L P
S V V N L P P V C R D S P F S N L P D L V L P
- - - - - - - - - - - - - - - - - - - - - - -
- - - - - - - - - - - - - - - - - - - - - - -
R L E P L S T E E R R N P L R S I P D F I P P
R V E P L Y T A E R D D A V N S L P D F V S P
R V E P F Y T A E R D D A L N S L P D F V S P
  .     L         R           S L P .     . P
```

```
 100                 110                 120
L A E I S I E K D V D G - - - - - - - F H P E
V E H S V R K W Q R - - - - - - - K M D P W S
L D M V Q D V P H T S S - - - - P E D C S G K
L N R L Q K R W D V P - - - - - - - - - - - -
V E H S A R E W Q R - - - - - - - K M D P W S
V E H S A R K W Q R - - - - - - - K T D P W S
- - - - - - - - - - - - - - - - - - - - - - -
V E L I K D N K F Y Q N N S W P R T I D C T G
V E H S A R K W Q R - - - - - - - K T D P W S
V K R T G K N W E V D W F G D G F Y L Y E P S
V A L C G K Q W D F D W F G E G V Q E L Q P S
- - - - - - - - - - - - - - - - - - - - - - -
- - - - - - - - - - - - - - - - - - - - - - -
P E K V G R Q W E F D W F D R A K V P L E P S
A E K A E N Q W D F D W F S R V K V P L Q P S
A E K A E N Q W D F D W F S R V K M P L Q P S
V E .         W     D W F                   S
```

```
                                           130                           140                           150
Ostreococcus tauri          N I G K L A M K G H E P L F V P C T P - - - - - - - - - K G C I E L L K R
Canis lupus                 L L A T L G A P V P S D L Q A Q R - - - - - - - - - - H P N T G Q I L G Y
Saccharmyces cerevisiae     L D Y K E L L K V P D P I N R T S Y Q F K R T G - - - L E G K I S G Y K E
Aspergillus nidulans        V N Y T D L Y E I A P T Q T R T I V R F T R E G - - - L E G R V T G Y H E
Bos taurus                  L L A T L G A P V P S D L Q A Q R - - - - - - - - - - H P T T G Q I L G Y
Homo sapiens                L L A V L G A P V P S D L Q A Q R - - - - - - - - - - H P T T G Q I L G Y
Pan troglodytes             - - - - - - - - - - - - - - - - - - - - - - - - - - - - - - - - - - - - -
Dictyostelium discoideum    M A W K D L L G I Q V S E P S S T I E L R V D N - - - V N H R L S M T E Y
Rattus norvegicus           L L A A L G T P V P S D L Q A Q R - - - - - - - - - - H P T T G H I L G Y
Selaginella moellendorfii   L A P L T I S P V W K P P F R R K E S E Y R T S E - - E Q G R V W V P E F
Physcomitrella patens       L P C L T I E P V W E P P Y T R G Q G D V R N G - - - H G V E D W I P D F
Brachypodium distachyon     - - - - M L V P A W E P P F R R R - P S L S P S - - - - Q E P Q V W D P E S
Oryza sativa                - - - - M L V P A W E P P F R R R R P P L S S S S S R Q E S Q V W D P E S
Glycine max                 L P R T M V V P V W E P P F R R S - - - N N G S - - - - V K G I W E P K F
Arabidopsis lyrata          L P R S V V V P T W E L P F R R Q - - - K V D T - - - - E N G A W E P K S
Arabidopsis thaliana        L P R S V V V P T W E L P F R R Q - - - K E D T - - - - E N G A W E P K S
                            L         . . P V           R                     S R         .   .

                                           190                           200                           210
Ostreococcus tauri          - - - - A M L L M K E N C T V T V V H S K T K D - - - - - - - - - - - - P
Canis lupus                 P I - - S Q S L W G N P T Q Y P F W P G G M D E - - - - - - - - P T I T D
Saccharmyces cerevisiae     H N - - Q N S V R G S T A Q L P F T P G G I P M K - - - S V K T G S E Q N
Aspergillus nidulans        G R - - A D F V R G A A G F F P F A P G G L D G - - - - - V E A I A E M E
Bos taurus                  P I - - S Q S L W G N P T Q Y P F W P G G M D E - - - - - - - - P S I T D
Homo sapiens                P A - - S Q S L W G N P T R Y P F W P G G M D E - - - - - - - - P T I T D
Pan troglodytes             - - - - - - - - - - - - - - - - - - - - - - - - - - - - - - - - - - - - -
Dictyostelium discoideum    K H N G T D F H R G D A S S F P F L P G G L D D I Q K K T N - - N N N I E
Rattus norvegicus           P A - - S Q S L W G N P T Q Y P F W P G G M D E - - - - - - - - P S I T D
Selaginella moellendorfii   N P - - A D F V R G S S S N Q P F R P G G V D L G - - - - - - - E K I I P
Physcomitrella patens       H P - - E D F V R G T T S N H P F R P G G F D L P Q - - - S S - G K T V P
Brachypodium distachyon     P P - - K D F V R G R V N S R P F R P G G M H D D T A E A A V L E K A F P
Oryza sativa                P A - - K D F V R G S V N S R P F R P G G L H D D A A A A A L E K A F P
Glycine max                 - - - - K D F V R G S I N N R P F R P G G L D D - - - - S R S L D R I L P
Arabidopsis lyrata          P P - - K D F L R G S V N N R P F R P G G L E D - - - - S Q S S E R V L P
Arabidopsis thaliana        P P - - K D F L R G S V N N R P F R P G G L E D - - - - S Q S S E R V L P
                            P   N G   D F . R G       .   P F   P G G   D .     A   S             . P

                                           250                           260                           270
Ostreococcus tauri          D W V K K G A V I I D V G - - - - - T N G V D D K T K - - - K A G Y R L V
Canis lupus                 P G F K K G V D F A P K D H S - T P A P G L L S L S H L L E P L D L G G G
Saccharmyces cerevisiae     E G M N R G I K P M D S P A E N E D Q N G Q F K E L K Q L N E I D N E L D
Aspergillus nidulans        N F G A E G G L L E I A P G C S R G L Q F E A T K T K E T A E G D E E V E
Bos taurus                  P G F K K G V D F A P K G H S - A P A A G L L S L S R L L E P L D L G G G
Homo sapiens                P G F K K G M D F A P K D C P - T P A P G L L S L S C L L E P L D L G G G
Pan troglodytes             - - - - - - M D F A P K D C P - T P A P G L L S L S C L L E P L D L G G G
Dictyostelium discoideum    P G M I E G L I I I E E K E K - - E K E K I E E N L F Y N Q E M E E E E E
Rattus norvegicus           P G F K K G V D F A P K - - - - A P V P G L L S L S R L L E P L D L S G G
Selaginella moellendorfii   P G F K R G L S N - L G V P E - P F T W S K K S D V S S - A E T F K E P E
Physcomitrella patens       P G F K H G I D - - F G L P E - P Y I V R Q G W D V T - - A E T S R S A E
Brachypodium distachyon     P G F G T G L D L G R L K E Y - N S H W K C F R D G E Q V E E Q P A S S S
Oryza sativa                P G F R K G L D L G N L K E Y - K S H W K C Y Q D G E R V D E Q S T S S S
Glycine max                 P S L K Q G L D F G M L K P Y - P C S W N V C K E A N - - S L K S S S D E
Arabidopsis lyrata          P S F K Q S L D L G D L M P Y - P Q T W S V Y E D Q S - - S H G N A S D E
Arabidopsis thaliana        P S F K Q S L D L G D L M P Y - P Q T W S V Y E D H S - - S H G N A S D E
                            P G F K . G . D   .   .                   .           L     .       .
```

```
  160                 170                 180
S G V E L S G K N A V V V G R S N I V G M P A
K E V L L E N T N L S A T T S L S L R R P P G
E V D L K E V A N A N A S N S L S I T R S I N
V T - - V P A A A A N A K N S T S L L R R P A
K E V L L E N T N L S S T T S L S L R R P P A
K E V L L E N T N L S A T T S L S L R R P P G
- - - - - - - - - - - - - - - - - - - - - - -
H E V P L N Q Q I T S T - N S T S L M R A W N
K E V L L E N T N L S A T T S L S L R R P P G
E Q I K P E Y G - - - S D A A S S M L R K P G
E Q V Q T E H A N L N S R N S S S V L R K P G
V Q L E M A Q V F D S G - T G G M V A R M P G
V Q M D M S D V F D S G - T G G I T P R M P G
E E V D V A D L T S G A V E S G P L P R T S G
V E V D L S E Q M Y G D Q D S G F F P R M V G
V E V D L S E Q M Y G D Q D S G F F P R M V G
  E V   L                 .   S . S .   R   P G

  220                 230                 240
K R V C R E A D V I V A A C G S - A E M V K K
L S T R - E E A E E E I D F E Q - D L L T V P
G S S T M A N A T K L L H K D G Q G L F D I P
S E A Q A A E R S K T G G K Q S - G L D R I I
L S T R - E E A E E E I D F E K - D L L T L P
L N T R - E E A E E E I D F E K - D L L T I P
- - - - - - - - - - - - - - - - - - - - - - -
K E E I D I D W L S F W K D P S - S L L N K P
L S T R - E E A E E E I D F E K - D L L T V P
E G A Q N G E W L A E V L E G K - P L Q T V A
A G A L N G D W L R E V L H G G - P L Q K V A
E G A R N G D W V R E L M S G G - P A Q I A P
E G A R N G D W V R E L M S G G - P A Q V N P
E G A S N G E W V H E I L N G G - P A Q T I P
E G V S S G Q W V Q E L L N G G - P A Q T V P
E G V S S G Q W V Q E L L N G G - P A Q T V P
    .     . . W .   E .     G   Q   L   T . P

  280                 290                 300
G - - - - - - - - - - - - - - - D V D Y A E V
D - - - - - - - - - - - - - - E D E T E A V G
I R I E - - - - - - - - - - A N E A K L K E E
H A L Q - - - - - - - - - - Q E E S D L H V E
D - - - - - - - - - - - - - - E D E S E A V G
D - - - - - - - - - - - - - - E D E N E A V G
D - - - - - - - - - - - - - - E D E N E A V G
E - - - - - - - - - - - - M E E E E I E Q E E
D - - - - - - - - - - - - - - E G E G E A A G
K K D S - - - - - - - - V L H Y E D L F R - -
V Q E S S V F N H A S V A L I D D S L F N N I
N Y - - - - - T M D K Y S V Q F D D L F K I A
N D - - - - - T M D K Y S V Q F D D L F K I A
K - - - - - - - L S G L S V Q F D D L F K K A
N S V C R S M S L V K L S I Q F D D L F K K A
N - - - - - - - - S S K L S I Q F D D L F K K A
                    K   S   Q   D . .       .
```

	310 320 330
Ostreococcus tauri	R - K
Canis lupus	Q - P G
Saccharmyces cerevisiae	- E K S
Aspergillus nidulans	R D - E D V
Bos taurus	Q - P G
Homo sapiens	Q - P G
Pan troglodytes	Q - P G
Dictyostelium discoideum	E E E E - M E E
Rattus norvegicus	- -
Selaginella moellendorfii	- K A
Physcomitrella patens	L R V V P V Q E L - D K E
Brachypodium distachyon	W E E D A V N N V F T E D D V Q Q L V R D E G S Y E I D E R K V D T L Q G
Oryza sativa	W E E D S D D K V P R E D H V Q Q L V G D E E T N D V D K Q N I S K L Q N
Glycine max	W D E D A V G D Q - - E D G - - - - - - - - - - - - - - H - - - - - L S E
Arabidopsis lyrata	W E E D T F S E L - - E R D G V - - - - - - - - - - F N H S Y H T A G S E
Arabidopsis thaliana	W E E D T F S E L - - E G D - - - - - - - - - - - - - - D - - H T A G S E
	E E D E D D V Q Q L V D E D

	370 380 390
Ostreococcus tauri	I C M L L S N T L D S G A P F A L A S R R - - - - - R P V R - - - - - A -
Canis lupus	R A S S L E D L V L K E T S T A I S P P E - - - P P K P V P Q - - - - E Q
Saccharmyces cerevisiae	A D N A D D A E I D E L L P I G I D F G R T K P V S K S V P V - - - K K E
Aspergillus nidulans	G E E D I D S L L P V E F P A L E P R A P L L S G V K Q R Q G - - - G K E
Bos taurus	R A S S L E D L V L K E A A T T V A P P E - - - P P K P P P Q - - - - E Q
Homo sapiens	R A S S L E D L V L K E A S T A V S T P E - - - A P E P P S Q - - - - E Q
Pan troglodytes	R A S S L E D L V L K E A S T A V S T P E - - - A P E P P S Q - - - - E Q
Dictyostelium discoideum	S S I S I D S I I S I D S S T S S S L L K F K K K E E E D P L S N - Q K M
Rattus norvegicus	R A S S L E D L V L K E A S T V V S T L E - - - P L K P P P Q - - - - E Q
Selaginella moellendorfii	D E V V L K D E V E P E S E V D K I L A T E T V E A A P V P K A E A K Q E
Physcomitrella patens	Y E E V L L D E I L G D P K V V I K S A K A H L K M S P N - - - - - R E V
Brachypodium distachyon	A Q T D L D K M L S S E V Q D T H R E L S G S V D D K P A Q E - - - G M V
Oryza sativa	P Q S D L D Q M L L S S V K D T S R D S S G S G D G S M A K E - - - G K V
Glycine max	S E M S L D D I L S A D S E G S K L H L D G F N D E I G Q Q K - - - K E A
Arabidopsis lyrata	T D I T V L D E I L S S A K T A I L T D E A V T G N S D K Q L L - - K E G
Arabidopsis thaliana	T D V T V L D E I L S S A K T A I M S E E A V T G S S D K Q L R - - K E G
	. L . D . L . . . . P A E

	430 440 450
Ostreococcus tauri	T S R S L V Y T A P T G A G K S R - V A D A L L E E T L A S D G G G R A L
Canis lupus	Q W S F E P D V F Q K Q A I L H L E R H D S V F V A A H T S A G - - K T V
Saccharmyces cerevisiae	S W P F E L D T F Q K E A V Y H L E Q G D S V F V A A H T S A G - - K T V
Aspergillus nidulans	E W P F E L D T F Q K E A V Y H L E N G D S V F V A A H T S A G - - K T V
Bos taurus	Q W A F E P D V F Q K Q A I L H L E R H D S V F V A A H T S A G - - K T V
Homo sapiens	Q W A F E P D V F Q K Q A I L H L E R H D S V F V A A H T S A G - - K T V
Pan troglodytes	Q W A F E P D V F Q K Q A I L H L E R H D S V F V A A H T S A G - - K T V
Dictyostelium discoideum	V Y P F E L D S F Q K Q A I V H M E K G E S V F I S A H T S A G - - K T V
Rattus norvegicus	Q W A F E P D V F Q K Q A I L H L E Q H D S V F V A A H T S A G - - K T V
Selaginella moellendorfii	Q F P F E L D K F Q K E A I Y H L E K N E S V F V A A H T S A G - - K T V
Physcomitrella patens	S F P F K L D T F Q K E A I Y H L E R N E S V F V A A H T S A G - - K T V
Brachypodium distachyon	E F P F E L D K F Q K E A I Y Y L E K G E S V F V A A H T S A G - - K T V
Oryza sativa	E Y P F E L D K F Q K E A I Y Y L Q K G E S V F V A A H T S A G - - K T V
Glycine max	E F P F E L D A F Q K E A I Y Y L E K G E S V F V A A H T S A G - - K T V
Arabidopsis lyrata	E F P F E L D N F Q K E A I C C L E K G E S V F V A A H T S A G - - K T V
Arabidopsis thaliana	E F P F E L D N F Q K E A I C C L E K G E S V F V A A H T S A G - - K T V
	. P F E L D F Q K E A I H L E . . . S V F V A A H T S A G G G K T V

```
  340                 350                 360
V A S A I T P V P G G - - - - - - - V G P M T
I P R G D T V S A T P - - - - - C - S A S L A
A K S I S E E I M E E - - - - - A - T E E T T
M S D V E G G V K I G - - - - - E - D D E L S
G P R Q D T V S A S P - - - - - G - S V P L A
G P R G D T V S A S P - - - - - C - S A P L A
G P R G D A V S A S P - - - - - C - S A P L A
M E E M K E M K K E E - N I S S T L N T L S P
G P R G D A A S A S P - - - - - S - S T P L I
W E H Q M L E D D S E - - - - - V D D E D P I
A S A V Y L D A Q T E - - - - - N G H Q A M R
A S A T L T R P D T D K Q E F D V I G D V S E
A S E T I E N L D I E K Q K G G A Q G D V S E
V E T I T L E A E V G - - T T E V S S R A H E
S P K A E A E P E A K A S I S N E V S K G L E
S P K A E A E P D A K A S I S N E V S K G L E
.     .                             .
```

```
  400                 410                 420
F P T R F N F N H S F N R L L E C L S R V A R
W A I P V D V T S P V G D F Y R L I P Q P A F
W A H V V D L N H K I E N F D E L I P N P A R
W A H V V D V N K H I S N F H E L V P D M A R
W A I P V D V T S P V G D F Y R L I P Q P A F
W A I P V D A T S P V G D F Y R L I P Q P A F
W A I P V D A T S P V G D F Y R L I P Q P A F
W A F H E T - K E I F T P F A E L I T N P A I
W A V P V D V T S P V G D F Y R L I P Q P A F
W V V M D G D S G V A E R F L E L I P D M A I
W A V M E P V P N I S Q R F E E L V P D L A L
W A L A G G D E D I A T N F H R F V P D M A I
W A L V G G D E D I V T N F Y K L V P D M A I
W A I H E T S E Q I V D S F H E L V P D M A L
W A T K G D S Q D I A D R F Y E L V P D M A I
W A T K G D S Q D I A D R F Y E L V P D M A I
W A .   . D         .     F   E L . P     A
```

```
  460                 470                 480
V V L P Y V A L V R E R A M A L A K T L R A R
V A E Y A I A L A Q K H M T R T I Y T S P I K
V A E Y A I A M A H R N M T K T I Y T S P I K
V A E Y A I A L A S K H M T K A I Y T S P I K
V A E Y A I A L A Q K H M T R T I Y T S P I K
V A E Y A I A L A Q K H M T R T I Y T S P I K
V A E Y A I A L A Q K H M T R T I Y T S P I K
I A E Y A I A M A A K N M T R A I Y T S P I K
V A E Y A I A L A Q K H M T R T I Y T S P I K
V A E Y A F A L S A K H C T R A V Y T S P I K
V A E Y A F A L A A K Q C T R A V Y T S P I K
V A E Y A F A L A T K H C T R A V Y T A P I K
V A E Y A F A L A T K H C T R A V Y T A P I K
V A E Y A F A L A S K H C T R A V Y T A P I K
V A E Y A F A L A T K H C T R A V Y T A P I K
V A E Y A F A L A T K H C T R A V Y T A P I K
V A E Y A . A L A   K H . T R A . Y T S P I K
```

Species	490 / 500 / 510
Ostreococcus tauri	G I G V R A Y A G G E S E G W A L G - - - - - G D A R C - - A V T T I E K
Canis lupus	A L S N Q K F R D F R N T F G D V G - - L L T G D V Q L H P E A S C L I M
Saccharmyces cerevisiae	A L S N Q K F R D F K E T F D D V N I G L I T G D V Q I N P D A N C L I M
Aspergillus nidulans	A L S N Q K F R D F R T E F D D V - - G I L T G D V Q I N P E A S C L I M
Bos taurus	A L S N Q K F R D F R N T F G D V G - - L L T G D V Q L H P E A S C L I M
Homo sapiens	A L S N Q K F R D F R N T F G D V G - - L L T G D V Q L H P E A S C L I M
Pan troglodytes	A L S N Q K F R D F R N T F G D V G - - L L T G D V Q L H P E A S C L I M
Dictyostelium discoideum	A L S N Q K F R D F K N T F G D V G - - L I T G D V S V S P A S S C L V L
Rattus norvegicus	A L S N Q K F R D F R N T F G D V G - - L L T G D V Q L H P E A S C L I M
Selaginella moellendorfii	T I S N Q K Y R D F S E K F - D V G - - L L T G D V S I R P E A S C L I M
Physcomitrella patens	T I S N Q K F R D F G G K F - D V G - - L L T G D V S L R P E A S C L I M
Brachypodium distachyon	T I S N Q K Y R D F C G K F - D V G - - L L T G D V S I R P E A T C L I M
Oryza sativa	T I S N Q K Y R D F C G K F - D V G - - L L T G D V S I R P E A T C L I M
Glycine max	T I S N Q K Y R D L C G K F - D V G - - L L T G D V S L R P E A S C L I M
Arabidopsis lyrata	T I S N Q K Y R D F C G K F - D V G - - L L T G D V S I R P E A S C L I M
Arabidopsis thaliana	T I S N Q K Y R D F C G K F - D V G - - L L T G D V S I R P E A S C L I M
	. . S N Q K F R D F F G D V G I G L L T G D V . . P E A S C L I M

Species	550 / 560 / 570
Ostreococcus tauri	V D E L H M V S E D E R G G V L E G M L A R I - R H A T R S G R A R R G G
Canis lupus	F D E V H Y I N D A E R G V V W E E V L I M L P D H V S - - - - - - - - -
Saccharmyces cerevisiae	F D E V H Y V N D Q D R G V V W E E V I I M L P Q H V K - - - - - - - - -
Aspergillus nidulans	F D E V H Y V N D L E R G V V W E E V I I M L P E H V T - - - - - - - - -
Bos taurus	F D E V H Y I N D A E R G V V W E E V L I M L P D H V S - - - - - - - - -
Homo sapiens	F D E V H Y I N D V E R G V V W E E V L I M L P D H V S - - - - - - - - -
Pan troglodytes	F D E V H Y I N D A E R G V V W E E V L I M L P D H V S - - - - - - - - -
Dictyostelium discoideum	F D E V H Y L N D Y E R G V V W E E V I I M L P A H V K - - - - - - - - -
Rattus norvegicus	F D E V H Y I N D A E R G V V W E E V L I M L P E H V S - - - - - - - - -
Selaginella moellendorfii	F D E V H Y V N D A E R G V V W E E V I I M L P Q H V N - - - - - - - - -
Physcomitrella patens	F D E V H Y V N D V E R G V V W E E V I I M L P P H V N - - - - - - - - -
Brachypodium distachyon	F D E V H Y V N D A E R G V V W E E V I I M L P K H I N - - - - - - - - -
Oryza sativa	F D E V H Y V N D A E R G V V W E E V I I M L P K H I N - - - - - - - - -
Glycine max	F D E V H Y V N D V E R G V V W E E V I I M L P R H I N - - - - - - - - -
Arabidopsis lyrata	F D E V H Y V N D V E R G V V W E E V I I M L P R H I N - - - - - - - - -
Arabidopsis thaliana	F D E V H Y V N D V E R G V V W E E V I I M L P R H I N - - - - - - - - -
	F D E V H Y V N D . E R G V V W E E V I I M L P H V R S G R A R R G G

Species	610 / 620 / 630
Ostreococcus tauri	- - - - A E T Y V G - - T Y R P V E L R E H V V R E D - - - - - - G V F V
Canis lupus	R L K R R Q I Y V I S T V A R P V P L - E H Y L F T G N S P K T Q G E L F
Saccharmyces cerevisiae	R T K Q K N I Y V I S T P K R P V P L - E I N I W A K - - - - - - K E L I
Aspergillus nidulans	R T K K K D I Y V I S T A K R P V P L - E H Y L W A G - - - - - - K D K Y
Bos taurus	R L K R R Q I Y V I S T V A R P V P L - E H Y L F T G N S P K T Q G E L F
Homo sapiens	R L K R R Q I Y V I S T V T R P V P L - E H Y L F T G N S S K T Q G E L F
Pan troglodytes	R L K R R Q I Y V I S T V T R P V P L - E H Y L F T G N S S K T Q G E L F
Dictyostelium discoideum	R T K K M P I Y V I G T L K R P V P L - E H F I H T P S - - - - - N D L F
Rattus norvegicus	R L K R R Q I Y V I S T V A R P V P L - E H Y L F T G N S P K T Q G E L F
Selaginella moellendorfii	R T K Q K K I Y V T G T T K R P V P L - E H C L F Y S - - - - - - G E L H
Physcomitrella patens	R T K R K L I Y V T G T M Q R P V P L - E H C I Y Y G - - - - - - G E L Y
Brachypodium distachyon	R T K Q K K I R V T S T N K R P V P L - E H C L F Y S - - - - - - G E T Y
Oryza sativa	R T K Q K K I H V T S T N K R P V P L - E H C L F Y S - - - - - - G E V F
Glycine max	R T K Q K E I R V T G T T K R P V P L - E H C L F Y S - - - - - - G E L Y
Arabidopsis lyrata	R T K Q K E I R V T G T T K R P V P L - E H C L F Y S - - - - - - G E L Y
Arabidopsis thaliana	R T K Q K E I R V T G T T K R P V P L - E H C L F Y S - - - - - - G E L Y
	R T K K I Y V . S T K R P V P L R E H L F N S P K T Q G E L .

```
   520                 530                 540
A A N A V S R A L E R G S - - F D E L R I V V
T T E I L R S M L Y S G S D V I R D L E W V I
T T E I L R S M L Y R G A D L I R D V E F V I
T T E I L R S M L Y R G A D L I R D V E F V I
T T E I L R S M L Y S G S D V I R D L E W V I
T T E I L R S M L Y S G S D V I R D L E W V I
T T E I L R S M L Y S G S D V I R D L E W V I
T T E I L R S M L Y K G A D L I R D I E W V I
T T E I L R S M L Y S G S D V I R D L E W V I
T T E I L R S M L Y K G A D L V R D I E W V V
T T E I L R S M L Y K G A D I I R D I E W V I
T T E I L R S M L Y R G A D I I R D I E W V I
T T E I L R S M L Y R G A D I I R D I E W V I
T T E I L R S M L Y R G A D I I R D I E W V I
T T E I L R S M L Y R G A D I I R D I E W V I
T T E I L R S M L Y R G A D I I R D I E W V I
T T E I L R S M L Y . G A D . I R D . E W V I
```

```
   580                 590                 600
P R I V C M S A T V N A R S L E R L A K W L D
- - I I L L S A T V P N - A L E - F A D W I G
- - F I L L S A T V P N - T Y E - F A N W I G
- - L I L L S A T V P N - T Y E - F A S W V G
- - I I L L S A T V P N - A L E - F A D W I G
- - I I L L S A T V P N - A L E - F A D W I G
- - I I L L S A T V P N - A L E - F A D W I G
- - I I L L S A T V A N - P L E - F A D W I G
- - I I L L S A T V P N - A L E - F A D W I G
- - L V L L S A T V P N - I R E - F A D W V G
- - L V L L S A T V P N - T F E - F A D W I G
- - I V L L S A T V P N - A V E - F A E W I G
- - I V L L S A T V P N - T V E - F A D W I G
- - I V L L S A T V P N - T I E - F A D W I G
- - F V L L S A T V P N - T F E - F A D W I G
- - F V L L S A T V P N - T F E - F A D W I G
P R I . L L S A T V P N R   . E R F A D W I G
```

```
   640                 650                 660
K T G - - - - - - - - E G L T R V R D A P R A
L L L D S R G A F H T K G Y Y A A V E A K K E
P V I N Q N S E F L E A N F R K H K E I L N G
K I V D S N K R F L E T G W K E A D N V I S G
L L L D S R G A F H T K G Y Y A A V E A K K E
L L L D S R G A F H T K G Y Y A A V E A K K E
L L L D S R G A F H T K G Y Y A A V E A K K E
K I V D S N R N F L M E G Y S N A Y N S L Y K
L L L D S R G A F H T K G Y Y A A V E A K K E
R I C A N E - T F L P L G V K A A K D A H L A
K I C E Q S - T F L P E G I K E A Q K A H L A
K V C E K D - M F L T Q G F R E A K D T F K M
K I C E K D - A F L T Q G Y R E A K E V F K K
K I C E S E - K F L P Q G L K A A K K E A S R
K V C E N E - V F I P K G I K D A K D S Q K K
K V C E N E - V F L S K G I K D A K D S Q K K
K . . . S   G   F L     G . .   A   . .   K
```

```
                                             670                 680                 690
Ostreococcus tauri          - - - - - - - - - - - - - - - - - - - - - - - - - - - - - - G - - G - - S
Canis lupus                 - - - - - - R M S K H A Q T - - - - - - - - - - F G A K Q P T H Q G - - -
Saccharmyces cerevisiae     - - - - - - E S A K G A P S K T D - - - - N G R G G S T A R G G R G G S N
Aspergillus nidulans        R D K I K A Q K A A E A Q A Q S Q - - - - A Q R G G Q Q G R G - R G Q P T
Bos taurus                  - - - - - - R M S K H A Q T - - - - - - - - - - F G A K Q P T H Q G - - -
Homo sapiens                - - - - - - R M S K H A Q T - - - - - - - - - - F G A K Q P T H Q G - - -
Pan troglodytes             - - - - - - R M S K H A Q T - - - - - - - - - - F G A K Q P T H Q G - - -
Dictyostelium discoideum    V D - - - - K N N D K N K K - - - - - - - - - - - - - - T T G Q H G - N Q
Rattus norvegicus           - - - - - - R M S K H A Q T - - - - - - - - - - F G A K Q P T H Q G - - -
Selaginella moellendorfii   - - - - - - K T A V K K G P - - - V A P T Q G G R G N - V Q G R G G - P G
Physcomitrella patens       - - - - - - K T T K Q A S A G S S V G S A S Q G R G G G T H G R G G G P A
Brachypodium distachyon     - - - - - - K N S N K L G V - - - - - - K P G S K S G T P A T R P G - T Q
Oryza sativa                - - - - - - K N S S K L G M - - - - - - K P G S K P G T T A V R A G - T Q
Glycine max                 - - - - - - K K N L T A G G - - - - - - G S G P K P G I S P G H D K - A R
Arabidopsis lyrata          - - - - - - K T S N A V S V - - - - - - A P K Q Y T G S S A H Q D G - N K
Arabidopsis thaliana        - - - - - - K N S N A V S V - - - - - - A P K Q Q M G S S A H Q D G - S K
                              D K I K A K   S   . A       S   V         G     G .         .   G G
```

```
                                             730                 740                 750
Ostreococcus tauri          - - - - - - - - - - - - - - - - - - - - - - - - - - V V A E L V G E V F V
Canis lupus                 - - - - - - - - - - - - - - - - - - - - - - - - - - V Y L S L L A S L R T
Saccharmyces cerevisiae     - - S R G A G A I G S N K R K - F F T Q D G P S K K T W P E I V N Y L R K
Aspergillus nidulans        P A N R G T G N I A R T G R G G G R T T A A Q D K T I W V Q L V G H L R K
Bos taurus                  - - - - - - - - - - - - - - - - - - - - - - - - - - V Y L S L L A S L R T
Homo sapiens                - - - - - - - - - - - - - - - - - - - - - - - - - - V Y L S L L A S L R T
Pan troglodytes             - - - - - - - - - - - - - - - - - - - - - - - - - - V Y L S L L A S L R T
Dictyostelium discoideum    - - - - - - - - - - - - - - - - - - - - - - - - - - - W T R L I G L L K E
Rattus norvegicus           - - - - - - - - - - - - - - - - - - - - - - - - - - V Y L A L L A S L R T
Selaginella moellendorfii   - - K V I P E - - - E K N S R G G - - P W R S E T S Q W Y G L I N V L S K
Physcomitrella patens       T S K Q V A Q G A I Q A A M R G G G T G W R S E T S Q W Y T L I N N L N S
Brachypodium distachyon     K H H H T N S G A A A I Q - Q S S S G S K R S E S S F W M P L I N N L L K
Oryza sativa                K H H H A S S S A A A V Q - Q S T S G P R R S E S S F W M P L I N N L L K
Glycine max                 F Y G T G R G Y Q N N G N G Q S N W E L R R A D A S M L L M L I N K L S K
Arabidopsis lyrata          S A K D V G K S S Y S G N S Q N N G A F R R S A A S N W L L L I N K L S K
Arabidopsis thaliana        S V K D V G K S S Y S G N S Q N N G A F R R S A A S N W L L L I N K L S K
                                                        Q             R S     S   W .   L .     L
```

```
                                             790                 800                 810
Ostreococcus tauri          A G - I F H R H P A A M S L R E R F V E R L F D A C E G - - - - A P D V D
Canis lupus                 T S - L D L T T S S E K S E I H L F L Q R C L A R L R G S D R Q L P Q V L
Saccharmyces cerevisiae     E G - I N F C N N K E K S Q I H M F I E K S I T R L K K E D R D L P Q I L
Aspergillus nidulans        S N - Q D F C N A S E K S L I H M F I E K S L T R L K P E D R I L P Q I L
Bos taurus                  T S - L D L T T S S E K S E I H L F L Q R C L A R L R G S D R Q L P Q V L
Homo sapiens                T S - L D L T T S S E K S E I H L F L Q R C L A R L R G S D R Q L P Q V L
Pan troglodytes             T S - L D L T T S S E K S E I H L F L Q R C L A R L R G S D R Q L P Q V L
Dictyostelium discoideum    G G H L V L T S N S E K N I I K I F I E E S L A R L R P E D K D L P Q I H
Rattus norvegicus           T S - L D L T T S S E K S E I H L F L Q R C L A R L R G S D R Q L P Q V L
Selaginella moellendorfii   T G - S D L T T S T E K G V I R V F C N K A F S R L K G T D R Q L P Q V L
Physcomitrella patens       S S - I D L T I Q S E K S E I Y M F C Q K A F S R L K G S D R R L P Q V V
Brachypodium distachyon     F G - A D L T S N S D K S E I R V F C D K A F S R L K G S D R N L P Q V V
Oryza sativa                F G - A D L T S N S E K S E I R L F C D K A F S R L K G S D R N L P Q V V
Glycine max                 T G - T D L T S S S E K S E I R L F C D K A F S R L K G S D K N L P Q V V
Arabidopsis lyrata          T G - T D L T S S S E K S E I R L F C D K A F S R L K G S D R N L P Q V L
Arabidopsis thaliana        T G - T D L T S S S E K S E I R V F C D K A F S R L K G S D R N L P Q V L
                            T G H . D L T . S S E K S E I . . F . . K     . R L K G S D R . L P Q V L
```

```
   700                 710                 720
E R E R E - - - L G - - - - - - - - - - - - -
G P A Q D - - - R G - - - - - - - - - - - - -
T R G G R G - G R G N S T R G G A N R G - G -
G R G A P - - - R G N A Q R G G A P R G R G Q
G P A Q D - - - R G - - - - - - - - - - - - -
G P A Q D - - - R G - - - - - - - - - - - - -
G P A Q D - - - R G - - - - - - - - - - - - -
A S F A S V S K T G - - - - - - - - - - - - -
G P A Q D - - - R G - - - - - - - - - - - - -
G R G G - - - - R G - - N - - - - - - - - - -
G R G G S M P G R G G D N Q G K V G G R K G P
G R N P D T S S R G K D Q - - - - - - - K H P
G R N P D T S S R G R D Q - - - - - - - K N P
V Q K R E N T S H T K H H - - - - - - - G A N
S Q K H E A H S R G K Q N - - - - - - - K H S
S Q K H E A H S R G K Q N - - - - - - - K H S
G   .   .     S R G     R G G A   R G K
```

```
   760                 770                 780
H G - H S T L V F C A S K A Q C S N V A K R L
R A Q L P V V V F T F S R G R C D E Q A S G L
R E L L P M V V F V F S K K R C E E Y A D W L
E N L L P G C I F V F S K K R C E E N A D S L
R A Q L P V V V F T F S R G R C D E Q A S G L
R A Q L P V V V F T F S R G R C D E Q A S G L
R A Q L P V V V F T F S R G R C D E Q A S G L
K Q Q L P V I V F S F S K N K C Q E Y A Q S L
R A Q L P V V V F T F S R G R C D E Q A S G L
K N L L P V V V F C F S K S R C D Q S A D S L
K G L L P V V V F C F S K N R C D Q S A D S L
K S L V P V V I F C F S K N R C D R S A D S M
K S L V P V V I F C F S K N R C D R S A E S M
K S L L P V V I F C F S K N R C D K S A D S L
K S L L P V V V F C F S K N Y C D R C A D A L
M S L L P V V V F C F S K N Y C D R C A D A L
.   L L P V V V F   F S K   R C D     A .   L
```

```
   820                 830                 840
L V Q A I L S - G I A W H H A G L T T A E K N
H M S E L L H R G L G V H H S G I L P I L K E
K T R S L L E R G I A V H H G G L L P I V K E
R L R E L L S R G I A V H H G G L L P I M K E
H M S E L L H R G L G V H H S G I L P I L K E
Q M S E L L N R G L G V H H S G I L P I L K E
H M S E L L N R G L G V H H S G I L P I L K E
Q I K D F L E R G I G I H H G G L L P I V K E
H M S E L L R R G L G V H H S G I L P I L K E
R I E E L L K R G I G V H H A G L L P I V K E
R V Q E L L K R G I G V H H A G L L P I V K E
G I Q S L L R R G I G V H H A G L L P I V K E
G I Q S L L R R G I G V H H A G L L P I V K E
R V Q N L L R R G I G V H H A G L L P I V K E
R V Q S L L H R G I G V H H A G L L P I V K E
R L Q S L L H R G I G V H H A G L L P I V K E
. .   . L L . R G I G V H H . G L L P I V K E
```

	850 860 870
Ostreococcus tauri	V I E E G F R D G A I L A L T C T T T L A A G V N L P T R R - - - - T I -
Canis lupus	I V E M L F S R G L V K V L F A T E T F A M G V N M P A R T - - - - V V F
Saccharmyces cerevisiae	L I E I L F S K G F I K V L F A T E T F A M G L N L P T R T - - - - V I F
Aspergillus nidulans	I V E I L F A K S L V K I L F A T E T F A M G L N L P T R T - - - - V V F
Bos taurus	I V E M L F S R G L V K V L F A T E T F A M G V N M P A R T - - - - V V F
Homo sapiens	I V E M L F S R G L V K V L F A T E T F A M G V N M P A R T - - - - V V F
Pan troglodytes	I V E M L F S R G L V K V L F A T E T F A M G V N M P A R T - - - - V V F
Dictyostelium discoideum	L V E I L F S K S L V K V L F A T E T F A M G V N M P A K T - - - - V V Y
Rattus norvegicus	I V E M L F S R G L V K V L F A T E T F A M G V N M P A R T - - - - V V F
Selaginella moellendorfii	V V E M L F C R G V I K V L F S T E T F A M G V N A P A R T - - - - V A F
Physcomitrella patens	V V E M L F C R G V I K V L F S T E T F A M G V N A P A R T - - - - V A F
Brachypodium distachyon	V V E M L F C R G V I K V L F S T E T F A M G V N A P A R T - - - - V V F
Oryza sativa	V V E M L F C R G V I K V L F S T E T F A M G V N A P A R T L K D L V V F
Glycine max	V V E M L F C R G V I K V L F S T E T F A M G V N A P A R T - - - - V V F
Arabidopsis lyrata	V V E M L F C R G V I K V L F S T E T F A M G V N A P A R T - - - - V V F
Arabidopsis thaliana	V V E M L F C R G V I K V L F S T E T F A M G V N A P A R T - - - - V V F
	. V E M L F R G . I K V L F T E T F A M G V N . P A R T L K D L V V F

	910 920 930
Ostreococcus tauri	G R A G R K G Q S D V G E S F L I V M K - - - K D A V A W A Q N L V C G D
Canis lupus	G R A G R R G L D P T G T V I L L C K G - - R V P E M A D L H R M M M G K
Saccharmyces cerevisiae	G R A G R R G L D S T G T V I V M A Y N - - S P L S I A T F K E V T M G V
Aspergillus nidulans	G R A G R R G L D T V G Y V I I V S A G R D E A P P A G A L R K M I L G D
Bos taurus	G R A G R R G L D P T G T V I L L C K G - - R V P E M A D L H R M M M G K
Homo sapiens	G R A G R R G L D P T G T V I L L C K G - - R V P E M A D L H R M M M G K
Pan troglodytes	G R A G R R G L D P T G T V I L L C K G - - R V P E M A D L H R M M M G K
Dictyostelium discoideum	G R A G R R G L D K V G T V I I T A W K - - D M P D S S S Y S S M I L G Q
Rattus norvegicus	G R A G R R G L D P T G T V I L L C K G - - R V P E M A D L H R M M M G K
Selaginella moellendorfii	G R A G R R G L D T V G T V I V M C W D - - D I P D E G D L R R L L T G K
Physcomitrella patens	G R A G R R G L D K V G T V V I M C W D - - D I P E E G D L K R L L T G R
Brachypodium distachyon	G R A G R R G L D T I G T V M I M C R D - - E I P E E S D L K N L I V G K
Oryza sativa	G R A G R R G L D N I G T V I V M C R D - - E I P E E S D L K N L I V G K
Glycine max	G R A G R R G L D K I G T V I L M C R D - - E L P E E S D L E P V I V G S
Arabidopsis lyrata	G R A G R R G L D K T G T V V V M C R D - - E V P D E S D L R R V I V G S
Arabidopsis thaliana	G R A G R R G L D K T G T V V V M C R D - - E V P D E S D L R R V I V G S
	G R A G R R G L D G T V I . C . R D . . P E . D L . R . . G .

	970 980 990
Ostreococcus tauri	A P P T A E Q K K F Y L E S I A C G I L K T K E D A G E - - - - - - - - -
Canis lupus	L R V E D M M K R S F S E F P S R K D S K A H E Q A L D E L T K R L G A L
Saccharmyces cerevisiae	L R V E E M I K Y S F S E N A K E T L Q P E H E K Q I K V L Q E E L Q T I
Aspergillus nidulans	L K I E E M I K R S F S E N A T Q A L L P E H E K Q V Q L S E A S L A K I
Bos taurus	L R V E D M M K R S F S E F P S R K D S K A H E Q A L A E L T K K L G A L
Homo sapiens	L R V E D M M K R S F S E F P S R K D S K A H E Q A L A E L T K R L G A L
Pan troglodytes	L R V E D M M K R S F S E F P S R K D S K A H E Q A L A E L T K K L G A L
Dictyostelium discoideum	F K V E D M I K R S F S E F S T Q K D I P E L Q K A I E S L S I E Y Q A I
Rattus norvegicus	L R V E D M M K R S F S E F P S R K D S K A H E Q A L A D L T K R L G A L
Selaginella moellendorfii	L K V E D M L K R S F A E F H A Q R A L P E Q Q Q Q L L R Q E G E L S K M
Physcomitrella patens	L K V E D M L K R S F A E F H A Q R S L P E Q Q Q Q L L E R E G A L S K M
Brachypodium distachyon	L K V E D M L K R S F A E F H A Q K K L P E K E E L L L Q M L R Q P T K -
Oryza sativa	L K V E D M L K R S F A E F H A Q K N L P E K E K L L L Q M L R Q P T K -
Glycine max	L K V E D M L K R S F A E F H A Q K K L P E M Q Q L L K R K L N Q P T K -
Arabidopsis lyrata	L K V E D M L K R S F A E F H A Q K K L P E K Q Q L L M R K R S L P I K N
Arabidopsis thaliana	L K V E D M L K R S F A E F H A Q K K L P E K Q Q L L M I K R S L P T K -
	L K V E D M . K R S F E F Q K L P E . E Q . L L K

```
  880                     890                   900
- - I I P G Y I A G T R T P S M A Q Y K Q M A
D S M R K H D G S T F R D L L P G E Y V Q M A
S S I R K H D G N G L R E L T P G E F T Q M A
S G F R K H D G R G F R D L L P G E Y T Q M A
D S M R K H D G S T F R D L L P G E Y V Q M A
D S M R K H D G S T F R D L L P G E Y V Q M A
D S M R K H D G S T F R D L L P G E Y V Q M A
S H T R K H D G I N F R D L L P G E Y T Q M S
D S M R K H D G S T F R D L L P G E Y V Q M A
H G F R K H D G K S F R Q L Y P G E Y T Q M A
H S L R K H D G K T F R Q I L S G E Y T Q M A
D S L R K F D G K E H R K L L P G E Y I Q M A
D S L R K F D G K E H R K L L P G E Y I Q M A
D T L R K F D G K E F R Q L L A G E Y T Q M A
D A L R K F D G K E F R Q L L P G E Y T Q M A
D A L R K F D G K E F R Q L L P G E Y T Q M A
D S   R K H D G     F R   L L P G E Y   Q M A
```

```
  940                     950                   960
L P P L E S R L F P P S - - - - - - - R G R D
P S Q L Q S Q F R L T Y T M I L N L L R V D A
P T R L Q S Q F R L T Y N M I L N L L R I E A
P T K L R S Q F R L T Y N M I L N L L R V E A
P S Q L Q S Q F R L T Y T M I L N L L R V D A
P S Q L Q S Q F R L T Y T M I L N L L R V D A
P S Q L Q S Q F R L T Y T M I L N L L R V D A
P S K L N S Q F R L T Y N M I L N L L R V Q D
P S Q L Q S Q F R L T Y T M I L N L L R V D A
A T K L E S Q F R L T Y T M I L N L L R V E E
P T K L E S Q F R L T Y S M I L N L L R V E D
P T R L E S Q F R L T Y T M I L H L L R V E E
P T R L E S Q F R L T Y T M I L H L L R V E E
A T R L E S Q F R L T Y I M I L H L L R V E E
A T R L E S Q F R L T Y I M I L H L L R V E E
A T R L E S Q F R L T Y I M I L H L L R V E E
P T . L   S Q F R L T Y T M I L N L L R V E .
```

```
 1000                    1010                  1020
- - - - - - - - - L A C R T F A W T S E D D Y
E - - - - - - - E P D T T G Q L V D L S E Y Y
E - - - - - - - Y K S C E I C D N D I E K F L
K - - - - - - - R E P C D I C D I D L V A C H
E - - - - - - - E P E V T G Q L V D L P E Y Y
E - - - - - - - E P D M T G Q L V D L P E Y Y
E - - - - - - - E P D M T G Q L V D L P E Y Y
E P - - - - - - - I Q C I L G E P D I E N Y Y
E - - - - - - - E P D V T G Q L A D L P E Y Y
A - - - - - - A T I E C I L G D P T I E D Y Y
N - - - - - - V Q I N C I L G E P S I E D Y F
- - - - - - - - T I E C I K G E P S I E E Y Y
- - - - - - - - T I E C I K G E P A I E E Y Y
- - - - - - - - A I E C L K G E P T I E E Y Y
I E A D L L I Y S S R C I K G E P A I E D Y Y
- - - - - - - - H I E C I K G E P A I E D Y Y
E   A D L L I       . C   .   .     I E . Y Y
```

```
                                              1030                1040                1050
Ostreococcus tauri            S S I L L R Q E V S - - - - - - - - - - - - L K Q I H D E G V V M V R R T
Canis lupus                   G W G E E L T E T R S L I Q R R I M E S V N G L K S L S A G R V V V V K N
Saccharmyces cerevisiae       E L M L A Y K E A T V N L M Q E M V K S P S I L H I L K E G R L V A F R D
Aspergillus nidulans          D A A I E Y E K L T S E L H V G L L A S P V G K R L F M P K R L V V Y R K
Bos taurus                    S W G E E L T E T R S Q I Q H R I I E S V N G L K S L S A G R V V V V K N
Homo sapiens                  S W G E E L T E T Q H M I Q R R I M E S V N G L K S L S A G R V V V V K N
Pan troglodytes               S W G E E L T E T Q H M I Q R R I M E S V N G L K S L S A G R V V V V K N
Dictyostelium discoideum      N M F S E A Q V L N E N V Q R T I L S S - N N Q Q Y F G D G R V I V L S I
Rattus norvegicus             S W A E E L T E T R N M I Q R R I M E S V N G L K S L S V G R V V V V K N
Selaginella moellendorfii     K L A S E A D M L G E S I Q E K V M N S R A A Q Q A L I P G R I V T V K T
Physcomitrella patens         S V Y L E Y D K L G D K I Q E A V M Q S R G G Q Q A L V A G R V V L V R N
Brachypodium distachyon       D M F L E A E K L R E Y I T E A I M Q L P A S Q Q F L G P G R L V V V E S
Oryza sativa                  E M V L E A E A H R E S I T E A I M Q L P A S Q Q S L T P G R L V V V K S
Glycine max                   D L Y L E A E T Y S N Q I S E A I L Q S P S A Q Q F L N T G R V V I V K S
Arabidopsis lyrata            D M Y M E A N E Y N N K M S E A V M Q S P Y A Q N F L V P G R V V V M K S
Arabidopsis thaliana          D M Y M E A N E Y N N K M S E A V M Q S P Y A Q S F L V Q G R V V V M K S
                                  .   E .   E         I Q     I M   S     .       L     G R V V V V K

                                              1090                1100                1110
Ostreococcus tauri            Y R C S L P I A H A K K L R - - - - - - - - - - - - - - - - - - - - - - -
Canis lupus                   R V F T T L V L C D K P V S - - - - - - - - - - - E D P Q E R A P A T P D
Saccharmyces cerevisiae       C V I M T F T K P Y K L P N G - - - - - - - - - - - - - - - E P N H L I Y
Aspergillus nidulans          T P N I Q V L E I G K L S H R R - - - - - - - - - - - - - H P S D I L P F
Bos taurus                    R V F T A L V L C D K P V S - - - - - - - - - - - E D P R E R G P A S P D
Homo sapiens                  R V F T T L V L C D K P L S - - - - - - - - - - - Q D P Q D R G P A T A E
Pan troglodytes               R V F T T L V L C D K P L S - - - - - - - - - - - Q D P Q D R G P A T A E
Dictyostelium discoideum      Q Y L N S T I E R T F N I F S - - - - - - - - - - - - - - - - - - - - - -
Rattus norvegicus             R V F T T L V L C D K P A V S - - - - - - - - - - E N P R D K G P A T P D
Selaginella moellendorfii     M S I V L T L Y R G A V P A S - - - - - - - A K L Q P P T K K L D Q D G G Y
Physcomitrella patens         L P I V L A I H R A P L P A T R G N P G L A A V T K A T N E L A K A G - Y
Brachypodium distachyon       Q Y V V F V L T G E C T S S A L A P N L S N Q N D N A Q G E V Q Q G Y M V
Oryza sativa                  Q Y V V L V L T G D C T S S A L A P D S S N Q N E K E A G D F K Q G Y F V
Glycine max                   M Y I V F V I K P D M P S S V D N A S S S G N M Q N K S G A F D Q G Y F V
Arabidopsis lyrata            Q Y V V L V T K S E I P P P E K N M V S I G - - - K K S S E P S Q G Y F I
Arabidopsis thaliana          Q Y V V L V I K S E I P P P E K N M V S I G - - - K K S S D P S Q G Y F I
                                  .     . .     .                   S                   .       .

                                              1150                1160                1170
Ostreococcus tauri            - - - - - - - S E L D D A L R N G L N L T S R T H L L F F C I S N - - - -
Canis lupus                   C D H T V A K L Q P G D V A T I T T K V L R V N G E K I L E D F S - - - K
Saccharmyces cerevisiae       - - - - - - - R N F P K F Q K T D F Y M E E V P V T A I E V I T K R K F A
Aspergillus nidulans          - - - - - - - P L P T R A A D M T L K V C K I P L S D L E C V T N T I V K
Bos taurus                    C D H T V A K L Q P G D V A A I T T K V L R L N G D K I L E D F S - - - K
Homo sapiens                  C D H T V V K L Q P G D M A A I T T K V L R V N G E K I L E D F S - - - K
Pan troglodytes               C D H T V V K L Q P G D M A A I T T K V L R V N G E K I L E D F S - - - K
Dictyostelium discoideum      - - - - - - L E L D Q E H G L R G Y K I Y S S N G H E I Q R I C T E K I V
Rattus norvegicus             C E H T V A K L Q P G D V A A I S T K V L R V N G E K I S E D F S - - - K
Selaginella moellendorfii     G V I R L T M P H Y G T V G G V S F L V A E T S G Q E F L S I S K E K I R
Physcomitrella patens         G V M K I S L P H Y G T A A G F G Y V V M E V D N Q G F L S L C K A K I R
Brachypodium distachyon       G V I K I K L P H K G D A S G M G F E V R A V E N K E I V S I C T S K I K
Oryza sativa                  G V I N I K L P Y K G D A S G M G F E V R A I E N K E I M N I C A S K I K
Glycine max                   G V I T I R L P Y S G S A C G M G Y E V R E V D S K E F L C I C S S K I K
Arabidopsis lyrata            V V I K I E L P Y H G V A A G V G Y E A K G F D N K E F L C I C D S K I K
Arabidopsis thaliana          V V I K I E L P Y H G V A A G V G Y E V K G F D N K E F L C I C D S K I K
                                V     .   L       G . . A .     .   V     .   .     I L   I       K I K
```

```
 1060                  1070                  1080
G - - - - - E N S T E W C S T - - P E G F A F
Q - - - - E Y H N T L G V I L Q V S S N S A S
P - - - - N D C L K L G F V F K V S L K D A V
- - - - - D G F R T A G I I V R E G V G G G A
Q - - - - E H H N A L G V I L Q V S S N S T S
Q - - - - E H H N A L G V I L Q V S S N S T S
Q - - - - E H H N A L G V I L Q V S S N S T S
G D D M T F K N Y T I G V I L S C N N T I Q K
E - - - - E H H N A L G V I L Q V S S N S T S
T I - - - Y P V P A L G V V L R G P S G T T K
S I - - - V P V P T L G V I V R A A T G N T K
K S - - - D D D H L L G V I V K N P S G S L K
Q S - - - D D D H L L G V I V K T P S A A L K
E S - - - A Q D H L L G V V V E T P S P T N K
G T - - - G I D N L L G V V L K G P S N T N R
G M - - - G I D N L L G I V L K G P S N T N R
    D M T         . L G V I L   .   S   .
```

```
 1120                  1130                  1140
- - - - - - - - - - - - - - - - - - - - - - -
V P Y P D D L V G F K L F L P - - - - - E G P
F P - - - - - - K A D G Y R R - - - - - - - -
L P - - - - - - R F R H L L H - - - - - - - -
V P Y P D D L V G F K L F L P - - - - - E G P
V P Y P D D L V G F K L F L P - - - - - E G P
V A Y P D D L V G F K L F L P - - - - - E G P
- - - - - - - - - - - - - - - - - - - - - - -
V P H P D D L V G F K L F L P - - - - - E G P
F I S K K N K N D D D E F A V F S G S K K S S
F I S K K G G D N G D D F F M G I G S R K G T
I P P K G K R G M D D E F F S A G S T R K S S
I P - K G K R S M E D E Y F S S V S T R K G S
M P - K S R R V V V D E Y S T S V S A R K G K
A P - K S K R G F E E E F Y T K P S S R K G S
A P - K S K R G F E E E F Y T K P S S R K G P
. P   K       .         F         S S R K G
```

```
 1180                  1190                  1200
- E S N F L A N N L N W H V W S D W L E H D S
R Q Q P K F K K D P P I A A V T T A V Q E L L
A P - - - - L G K V I K K D V A A L N E F N A
V G G P T W Y L N I K K E A I K F A D K E L S
R Q Q P K F K K D P P S A A V T T A V Q E L L
R Q Q P K F K K D P P L A A V T T A V Q E L L
R Q Q P K F K K D P P L A A V T T A V Q E L L
N I N T K A I K D G D Q D S I G I L Q Q Q L L
R Q Q P K F R K D P P L A A V T T A V Q E L L
V D A N R I L E E E S T M A I S A V L Q A L T
V D S A R L L E D N S P A A Y T S T M K E L L
I D Q F R L L E E P S K T V Y S T T V Q L L I
I D Q V R L L E D P S K T V Y S K T V Q M L I
I D R V G L L E D I S S S V Y S K T V Q L L M
I D Q V R L L E D G N K S A F S Q T V Q Q L L
I D Q V R L L E D G N K A A F S Q T V Q Q L L
.   Q   . . .   D           A . .   . V Q   L L
```

	1210 · 1220 · 1230
Ostreococcus tauri	V L H Q L G E - - - - - - - - K H L G V T R - - - - E Y A E H A Q Y R L G
Canis lupus	R L A Q A Y P - A G - P P T L D P V N D L Q L K D V S V V E G G L R A R K
Saccharmyces cerevisiae	E T N N I L D - - - - G K T L K E A I N I E K Q G L K I H Q I L L D R T N
Aspergillus nidulans	K L C A S W T - - - - S P I W D E M D W A R I K E L Q V R D I L E K R Q A
Bos taurus	R L A Q A H P - T G - P P T L D P V N D L Q L K D V S V V E G G L R A R K
Homo sapiens	R L A Q A H P - A G - P P T L D P V N D L Q L K D M S V V E G G L R A R K
Pan troglodytes	R L A Q A H P - A G - P P T L D P V N D L Q L K D M S V V E G G L R A R K
Dictyostelium discoideum	R L L E K Y P L P L G P P S I D P I S K L K L R S I E F V D Q F D K L Q N
Rattus norvegicus	R L A Q A Y P - A G - P P T L D P I N D L Q L K D V A V V E G G L R A R K
Selaginella moellendorfii	E L E R L Y P - A D - P P P L D P V K D L K L N D I D A V E K Y K K K Q A
Physcomitrella patens	Q L E K E Y P G Q D - P P A L D L L K D L K F T D F G V V E A Y R K Q Q A
Brachypodium distachyon	K A Q P D G H - K Y - P P A L D P I K D L K M K D M D Q V Q K Y H A Y N R
Oryza sativa	K E Q P D G N - K Y - P A A L D A I K D L K M K D M L L V E N Y Y A Y Q R
Glycine max	D L K S D G N - K Y - P P A L D P V K D L K L R D V K L V A T Y H K W T R
Arabidopsis lyrata	D L K S D G N - K Y - P P P L D P I K D L K L K D A E S V E T Y Y K W T S
Arabidopsis thaliana	D L K S D G N - K F - P P A L D P V K D L K L K D A E L V E T Y Y K W T N
	. L G P P L D P . D L . L K D . . V E .

	1270 · 1280 · 1290
Ostreococcus tauri	- - - - - - - - - - - - - E L L A N D D T I F D I E Q K W S - - - - L M S
Canis lupus	R E R M Q I Q K E M E R L R F L L S D Q S L L L L P E Y H Q R V E V L R T
Saccharmyces cerevisiae	F K A H V I K K K I E E L Y H L M S D Q N L S L L P D Y E K R L A V L K D
Aspergillus nidulans	H D E W Q V K E N I S Q L K Q L M S D Q N L Q L L P D Y E Q R I Q V L R D
Bos taurus	R E R M Q I Q K E M E R L R F L L S D Q S L L L L P E Y H Q R V E V L R T
Homo sapiens	R E R M Q I Q K E M E R L R F L L S D Q S L L L F P E Y H Q R V E V L R T
Pan troglodytes	R E R M Q I Q K E M E R L R F L L S D Q S L L L L P E Y H Q R V E V L R T
Dictyostelium discoideum	K H K H D I K T K M N E Y K H T S S D E N L Q L M P E F Q I R L K I L E T
Rattus norvegicus	Q E R M Q I Q K E M E R L R F L L S D Q S L L L L P E Y H Q R V E V L R T
Selaginella moellendorfii	K N R Q L L R D R V D K L K F D V S D N A L Q Q M P E F Q R R M D V L Q D
Physcomitrella patens	K N Q H I L K E R V S Q L K Y E L S D A A L Q Q M P D F G K R I E V L Q A
Brachypodium distachyon	K E Q K V Y K T Q M D E L K Y Q M S D E A L Q Q M P Q F Q G R I D V L K E
Oryza sativa	K E Q K V Y K D Q L N D L K Y E M S D E A L Q Q M P E F Q G R I D V L K E
Glycine max	K E I K K H K E E V Y A L Q F Q M S D E A L K Q M P D F Q G R I D V L K Q
Arabidopsis lyrata	R E I K K H K T D L K D L E F Q M S D E A L L Q M P A F Q G R I D V L K N
Arabidopsis thaliana	R E I K K H K T D L K D L E F Q M S D E A L L Q M P A F Q G R I D V L K N
	. E . K L . F S D L P . . R . . V L .

	1330 · 1340 · 1350
Ostreococcus tauri	A T T A G M A S V L A N - - - - - - - E C G W S A L A G L M L N - - - - -
Canis lupus	H E L L L T E L M F D N A L S A L R P E E I A A L L S G L V C Q S P G - -
Saccharmyces cerevisiae	Y E L V L T E L I L D N F L G S F E P E E I V A L L S V F V Y E G K T R E
Aspergillus nidulans	D E L V L T E L I L E N V L A E Y E P E E I V A L L S A F V F Q E K T - -
Bos taurus	H E L L L T E L M F D N A L S T L R P E E I A A L L S G L V C Q S P G - -
Homo sapiens	H E L L L T E L M F D N A L S T L R P E E I A A L L S G L V C Q S P G - -
Pan troglodytes	H E L L L T E L M F D N A L S T L R P E D I V A L L S G L V C Q S P G - -
Dictyostelium discoideum	E E L I I P E L I F E N A F L M L E P S E I V S V L S C L I F Q E K D - -
Rattus norvegicus	H E L L L T E L M F D N A L S A L R P E E I A A L L S G L V C Q S P G - -
Selaginella moellendorfii	D E L I A A E C L F D N Q L A D L N A A E S I A L L S S L V F Q Q R E - -
Physcomitrella patens	D E L I A T E C L F D N Q L G D L T P A E A V A L L S S L V F Q Q K D - -
Brachypodium distachyon	E E L I S T E C L F E N Q L D D L E P E E A V A I M S A F V F Q Q R N - -
Oryza sativa	E E L I S T E C L F E N Q L D D L E P E E A V A I M S A L V F Q Q R N - -
Glycine max	E E L I C T E C L F E N Q M D E L E P E E A V A I M S A F V F Q Q K N - -
Arabidopsis lyrata	E E L I C T V C L F E N Q F E E L E P E E A V A I M S A F V F Q Q K N - -
Arabidopsis thaliana	E E L I C T V C L F E N Q F E E L E P E E A V A I M S A F V F Q Q K N - -
	. E L . . T E . F . N L L E P E E . V A L L S . L V F Q . R E

```
 1240                1250                1260
V S A E S R A V R S R H E R L A A A H L L S -
L E E L I W G A Q C V H S P R F S A Q Y V K L
I R D E I F K L K S I K C P N L S Q H I V P K
Q A A I T Q S C R C L Q C P S F M K H F E M Q
L E E L I R G A Q C V H S P R F P A Q Y L K L
L E E L I Q G A Q C V H S P R F P A Q Y L K L
L E E L I Q G A Q C V H S P R F P A Q Y L K L
I Q K L I P T S K C N N C P K L S N H Y T I T
L E E L I R G A Q C V H S P R F P A Q Y V K L
I T E L M A Q N K C H R C P K L Q E H Y S I I
L L E I M A Q N K C H K C P K L Q E H Y T L V
L L E K M S E N K C H G C I K L K E H K S L M
L L Q K M S E N K C H G C I K L K E H I A L M
L L E K M S Q N Q C H G C I K L E E H L K L A
L L Q K M S M N K C H G C V K L E E H M K L A
L L Q K M S M N K C H G C V K L E E H M K L A
L   E .         . C   . C P . L     H .     .
```

```
 1300                1310                1320
N G A I D - - - - - - - R G K I Q S L Q N M A
L G Y V D E A G T V K L A G R V A C A M S S -
T E F I D Q N H N V L L K G R V A C E I N S G
L G F I D E Q S R V Q L K G K V A C E I H S A
L G Y V D E A G T V K L A G R V A C A M S S -
L G Y V D E A G T V K L A G R V A C A M S S -
L G Y V D E A G T V K L A G R V A C A M S S -
L G Y I D G E N N V M V K G K V S R E V N T C
L G Y V D E A G T V K L A G R V A C A M S S -
V G C I D S E L I V Q L K G R V T C E F N T G
V E C I D A E L V V Q L K G R V A C E L N S C
I Q Y V D S D L V V Q L K G R V A C E M N S G
I H Y I D S D L V V Q L K G R V A C E M N S G
I G C I D E D L V V Q M K G R V A C E M N S G
I G C I D D D L V V Q I K G R V A C E M N S G
I G C I D D D L V V Q I K G R V A C E M N S G
. G . I D .   .   V Q L K G R V A C E M N S G
```

```
 1360                1370                1380
- - - - - V S E E L Q A G A R R E L L P L M R
D P G E Q L P S T L K Q G V E R V R A V A R R
E E P P I V T P R L A K G K Q R I E E I Y K K
E N V P T L T P R L E K G K E A I I R I A E K
D P G D Q L P S T L K Q G V E R V R T V A K R
D A G D Q L P N T L K Q G I E R V R A V A K R
D A G D Q L P N T L K Q G I E R V R A V A K R
A I E P S L T P R L I Q A R D N L I K I N E K
D P G D Q L P S T L K Q G V E R V K A V A K R
T S E P V L T E K L A A A K T R L Y N T A L Q
A S E P V L T E R L E Q A R D R L Y H T A I R
A S E P S L T P K L A D A K K R L Y D T A I R
T S E P S L T P K L A D A R K R I Y D T A I R
T S E P S L T P K L S E A K H R L Y Q T A I R
T S A P S L T S K L A K A K Q R L Y D T A I R
T S A P T L T P K L A K A K Q R L Y D T A I R
      P   L T   . L     . .   R .       A   R
```

	1390 / 1400 / 1410
Ostreococcus tauri	L D G M T G A R A R S - - - - - - - L Y N A G - F K T P T I I A S L A T E
Canis lupus	I G E V Q V A C G L N - - - Q T V E E F V G E - L N F G L V E V V Y E W A
Saccharmyces cerevisiae	M L S V F N T H Q I P - L T Q D E A E F L D R - K R F A M M N V V Y E W A
Aspergillus nidulans	V N D L Q I Q Y Q V I Q S S E D S N D F A S Q - P R F G L A E V V Y E W A
Bos taurus	I G E V Q A A C G L N - - - Q T V E E F V G E - L N F G L V E V V Y E W A
Homo sapiens	I G E V Q V A C G L N - - - Q T V E E F V G E - L N F G L V E V V Y E W A
Pan troglodytes	I G E V Q V A C G L N - - - Q T V E E F V G E - L N F G L V E V V Y E W A
Dictyostelium discoideum	L C Q L E I D H G L Q - - - - V T L E E K E K I L K F G L M E V T Y E W A
Rattus norvegicus	I G E V Q V A C G L N - - - Q T V E E F V G E - L N F G L V E V V Y E W A
Selaginella moellendorfii	L G D L Q V S H G L V - - - S H A E D Y A R D A L H F G L M E V V Y E W A
Physcomitrella patens	L G N V Q K S F D L S - - - L D P E D Y A R A N L K F G L M E V V Y E W A
Brachypodium distachyon	L G Q L Q K H H E V P - - - V D P E E Y A R D N L K F G L V E V V Y E W A
Oryza sativa	L G K L Q R E F K V P - - - V D P E E Y A R D N L K F G L V E V V Y E W A
Glycine max	L G E L Q A H F N L P - - - I N P A E Y A Q E N L K F G L V E V V Y E W A
Arabidopsis lyrata	L G E L Q A Q Y N L Q - - - I D P E E Y A Q E N L K F G L V E V V Y E W A
Arabidopsis thaliana	L G E L Q A Q Y N L Q - - - I D P E E Y A Q E N L K F G L V E V V Y E W A
	L G . . Q . L Q E E . . . N L . F G L V E V V Y E W A

	1450 / 1460 / 1470
Ostreococcus tauri	A G L D Q A M R T T - - A W R V A - - T S L A K N A R E V S V M E A R A A
Canis lupus	Q R L A E M C R S L R G A A R L V G E P V L G A K M E T A A T L L R R D I
Saccharmyces cerevisiae	T W L D E I C R E V K T A S I I I G N S T L H M K M S R A Q E L I K R D I
Aspergillus nidulans	T R L D E T C R E V R N A A K L V G D P T L Y A K M Q H A Q E L I K R D V
Bos taurus	Q R L A E M C R S L R G A A R L V G E P V L G A K M E T A A T L L R R D I
Homo sapiens	Q R L A E M C R S L R G A A R L V G E P V L G A K M E T A A T L L R R D I
Pan troglodytes	Q R L A E M C R S L R G A A R L V G E P V L G A K M E T A A T L L R R D I
Dictyostelium discoideum	T R I G E T C Q E V R N C A R I I G D T K L Y Q K M D E A I R L I K R D I
Rattus norvegicus	Q R L A E M C R S L R G A A R L V G E P V L G A K M E T A A T L L R R D I
Selaginella moellendorfii	V R L D E T C R E I K N A A R I M G D T T L F N K M D E A S N L I K R D I
Physcomitrella patens	V R L D E T C R E F R N A A R L I G D S T L F E K M E Q A S N A I K R D I
Brachypodium distachyon	V R L D E T C R E F R N A A S I M G N S A L F K K M E I A S N A I K R D I
Oryza sativa	V R L D E T C R E F R N A A S I M G N S A L H K K M E T A S N A I K R D I
Glycine max	V R L D E T C R E F K N A A A I M G N S A L C K K M E I A S N A I K R D I
Arabidopsis lyrata	V R L D E T C R E F K N A A A I M G N S A L H K K M D A A S N A I K R D I
Arabidopsis thaliana	V R L D E T C R E F K N A A A I M G N S A L H K K M D A A S N A I K R D I
	R L D E T C R E . R N A A R . . G . . . L K M E A L I K R D I

```
  1420                          1430                          1440
K L D K L V E A C L K S L S R S S - - - R - -
R G M P F S E L A G L S G T P E G L V V R C I
R G L S F K E I M E M S P E A E G T V V R V I
K G M S F N R I T D L T D V M E G T I V R T I
R G M P F S E L A G L S G T P E G L V V R C I
R G M P F S E L A G L S G T P E G L V V R C I
R G M P F S E L A G L S G T P E G L V V R C I
R G M P F N D I C K L T N V L E G T I V R A I
R G M P F S E L A G L S G T P E G L V V R C I
K G T P F S T I C E M T D V S E G L V V R T I
K G T S F A D I C E I T N V P E G S I V R T I
K G T P F A D I C E L T D V S E G L I V R T I
K G T P F A D I C E L T D V S E G L I V R T I
K G T P F A D I C E L T D V P E G L I V R T I
K G T P F A E I C E L T D V P E G L I V R T I
K G T P F A E I C E L T D V P E G L I V R T I
K G   P F   E I C . L T   V P E G L I V R T I
```

```
  1480                          1490                          1500
L A N L E E - - - - - - - -
V F A A S L Y T Q - - - - -
V F A A S L Y L - - - - - -
I F A A S L Y M - - - - - -
V F A A S L Y T Q - - - - -
V F A A S L Y T Q - - - - -
V F A A S L Y T Q - - - - -
V F A S S L Y V V - - - - -
V F A A S L Y T Q - - - - -
V F A A S L Y V T G L V - -
V F A A S L Y V T G V P V I
V F S A S L Y V T G I - - -
V F A A S L Y V T G I - - -
V F A A S L Y I T G V - - -
V F A A S L Y V T G V - - -
V F A A S L Y V T G V - - -
V F A A S L Y . T G V   V I
```
